# Influence of Critical Parameters on Cytotoxicity Induced by Mesoporous Silica Nanoparticles

**DOI:** 10.3390/nano12122016

**Published:** 2022-06-11

**Authors:** Amirsadra Ahmadi, Moses Sokunbi, Trisha Patel, Ming-Wei Chang, Zeeshan Ahmad, Neenu Singh

**Affiliations:** 1 Leicester School of Allied Health Sciences, De Montfort University, The Gateway, Leicester LE1 9BH, UK; p2569486@alumni365.dmu.ac.uk (A.A.); moses.sokunbi@dmu.ac.uk (M.S.); p17197170@my365.dmu.ac.uk (T.P.); 2 Nanotechnology and Integrated Bioengineering Centre, Jordanstown Campus, University of Ulster, Newtownabbey BT37 0QB, UK; m.chang@ulster.ac.uk; 3 Leicester School of Pharmaceutical Sciences, De Montfort University, The Gateway, Leicester LE1 9BH, UK; zahmad@dmu.ac.uk

**Keywords:** nanotoxicology, cytotoxicity, mesoporous silica nanoparticles, toxicity, zeta potential, functionalisation, diameter size, incubation

## Abstract

Mesoporous Silica Nanoparticles (MSNs) have received increasing attention in biomedical applications due to their tuneable pore size, surface area, size, surface chemistry, and thermal stability. The biocompatibility of MSNs, although generally believed to be satisfactory, is unclear. Physicochemical properties of MSNs, such as diameter size, morphology, and surface charge, control their biological interactions and toxicity. Experimental conditions also play an essential role in influencing toxicological results. Therefore, the present study includes studies from the last five years to statistically analyse the effect of various physicochemical features on MSN-induced in-vitro cytotoxicity profiles. Due to non-normally distributed data and the presence of outliers, a Kruskal–Wallis H test was conducted on different physicochemical characteristics, including diameter sizes, zeta-potential measurements, and functionalisation of MSNs, based on the viability results, and statistical differences were obtained. Subsequently, pairwise comparisons were performed using Dunn’s procedure with a Bonferroni correction for multiple comparisons. Other experimental parameters, such as type of cell line used, cell viability measurement assay, and incubation time, were also explored and analysed for statistically significant results.

## 1. Introduction

Mesoporous silica nanoparticles (MSNs) are considered a powerful tool for use in biomedicine. They have received tremendous attention recently due to their tuneable pore size, surface area, size, surface chemistry, and thermal stability [1,2,3]. MSNs are used for their ability to overcome low bioavailability, solubility, and acute side effects [4]. The porous morphology of MSNs provides a large surface area and pore volume for drug loading and protects the loaded therapeutics from enzymatic and other disrupting processes that might cause low bioavailability in the loaded material [5].

MSNs can also be conjugated with targeting materials by a covalent bond to increase their specificity [6]. The drug release performance of the MSNs can also be tuned with the help of various fabrication materials that result in surface functionalisation of the MSNs to control the release and effect of loaded therapeutics [7]. Different functionalisation types have been utilized to control the burst release of loaded agents that are sensitive to temperature, light, magnetic field, ultrasound, electric field, pH, redox agents, reactive oxygen species (ROS), enzymes, glucose, adenosine triphosphate (ATP), cell membranes, or a combination of activators [8].

MSNs are generally believed to have good biocompatibility and low toxicity, improving their capability in various biomedical applications [9]. However, some studies have investigated the toxicity of MSNs dependent on functionalised groups, time of exposure, and size. Indeed, the toxicity of MSNs is primarily influenced by physicochemical features, such as diameter size, morphology, surface charge, and functionalised groups, that broaden their applicability but could adversely regulate biological interactions. 

Characterization of MSNs in terms of size, aggregation state, surface area, and surface chemistry can offer insight into the interaction between nanoparticle physicochemical properties and their biocompatibility. Size measurement of nanoparticles holds importance as changes in size lead to changes in surface area and their subsequent bioavailability [10]. Size measurement using DLS (Dynamic Light Scattering) is commonly used to determine the hydrodynamic size of nanomaterials and is conducted under experimental conditions, which gives insight into the agglomeration state of nanomaterials in experimental media [11]. Changes in surface charge lead to changes in the ability of nanoparticles to penetrate cell membranes, and zeta potential is measured instead of the surface charge as it measures the electrostatic potential between the nanoparticles’ surface charge and the ionic solution’s electrostatic charge, which is measured by laser Doppler velocimetry [12].

Other factors and experimental conditions, including time of exposure to MSNs, cell type studied, and the cytotoxicity assay used, need to be considered to obtain an accurate, comprehensive overview of the toxicological profile of MSNs. Experimental conditions could largely determine MSN–cell interaction, affect cellular uptake, and influence downstream toxicity interpretation and assessment [13,14,15]. Therefore, it becomes increasingly important to understand the interaction of physicochemical properties of MSNs with their toxicity profiles in the light of experimental conditions to achieve a desired biocompatible design that is safe to use in various MSN-based applications. 

The current study extracted data on diameter size, zeta-potential measurements, functionalisation type, in-vitro cell lines, viability measurement assays, and incubation time of exposure to MSNs from studies that contained “mesoporous silica nanoparticles” and “toxicity” keywords in their titles or abstracts. Additionally, publishing dates were filtered from the last five years. Cytotoxicity assays are relatively inexpensive, easy to measure, and cell viability is a crucial end-point indicator of toxicological evaluation and assessment. Therefore, the aim was to find any differences in the different physicochemical characteristics (diameter size, functionalisation groups, zeta potential) and experimental factors (cell lines, cell viability assays, time of exposure), which may be related to cytotoxicity. The study investigated differences in the physicochemical characteristics and experimental factors of different groups.

## 2. Materials and Methods

The present study used the PubMed database to review the available literature on the toxicity of MSNs observed on cell lines.

### 2.1. Criteria for Paper Selection

**Figure 1:**
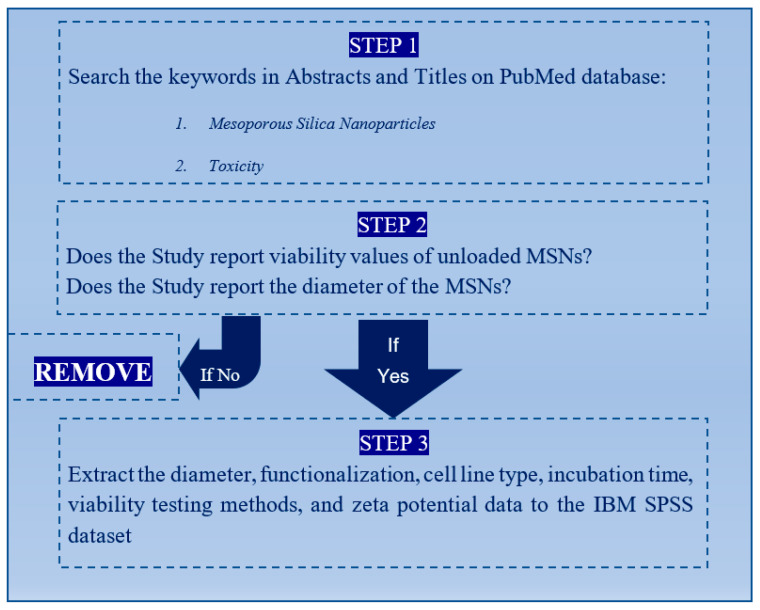
Schematic representation of the study selection procedure. This shows the flow chart of the paper selection process.

To collect data from different studies that fabricated MSNs and assessed their toxicities to analyse the effects of physicochemical properties on MSNs toxicity results, “mesoporous silica nanoparticles” and “toxicity” keywords were searched on PubMed in abstracts and titles of the articles with the publishing date specified as 2016 to 2021. A total of 192 articles was identified from the search on PubMed. Studies were first explored for those that reported the unloaded MSN’s diameter size and conducted in-vitro toxicity tests on cell lines. Furthermore, studies that reported in-vitro toxicity testing using MSNs, irrespective of whether these were functionalised or non-functionalised, were identified and selected. The next step entailed zeta-potential measurements from the resulting 45 studies. Figure 1 depicts the flow chart of the selection procedure used in the study. 

### 2.2. Statistical Tests

To investigate the effect of each parameter on the viability score of cell lines exposed to MSNs (from the selected studies), appropriate statistical tests need to be performed to determine statistically significant differences between viability scores among categories of one parameter/characteristic. Each parameter measurement had varying viability readings and behaved as the ordinal categorical independent variable. The viability score was considered the continuous dependent variable, as the viability varied in a continuous manner throughout the different critical parameters measured.

To acquire reliable results, tests for normality and the existence of outliers in the data were explored [16]. Due to non-normality and the presence of outliers, tests that analyse groups’ means, such as ANOVA and *t*-test, were not suitable (Tables S3–S8, Supplementary Materials). 

Kruskal–Wallis H test and Mann–Whitney U test were the suitable tests for this study due to non-normal data and the existence of outliers [17]. Kruskal–Wallis H test was used to compare the distributions and medians of more than two groups (replacing ANOVA), and the Mann–Whitney U test was used to compare the distribution and medians of two categorical groups (replacing *t*-test) [18]. Statistically significant results determined whether the variable had a significant effect on the outcome; in this case, viability. Further investigation of significant results was performed by post-hoc test to determine which categories of the variable caused significant differences in the results. Functionalisation, cell line type, incubation time, viability testing methods, diameter size, and zeta potential of MSNs were explored for significant differences.

**Figure 2:**
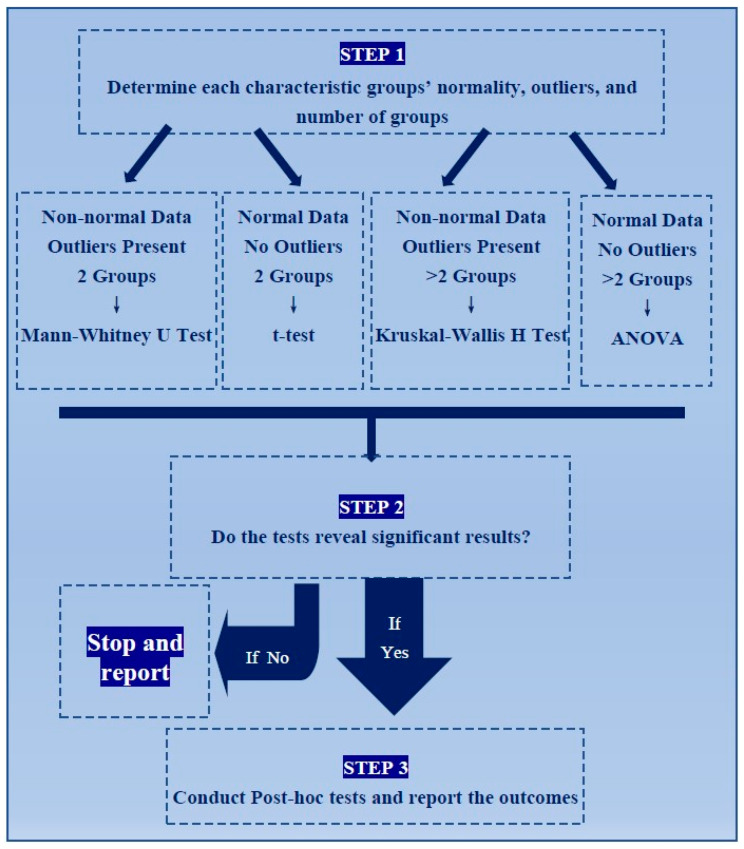
Schematic representation of analysis. The flow chart shows the steps and statistical decisions made in the analysis of data.

The 45 studies selected to be considered in the combinational study produced 1240 cases. Mean ranks of the study groups and a post-hoc test were carried out with IBM SPSS Statistics for Windows, version 26 (IBM Corp., Armonk, NY, USA), as shown in Tables S1 and S2 (Supplementary Materials). Figure 2 represents the procedure for analyses. IBM SPSS software was chosen as the statistical analysis tool for the present study. SPSS has been used in several MSN studies to determine significant differences in viability data between loaded and non-loaded nanoparticles (Table 1).

Based on the procedure as mentioned above for analysis, an inspection of the boxplot and normality tests revealed outliers and non-normality.

## 3. Results

### 3.1. Differences between MSN Sizes Based on Viability

**Figure 3:**
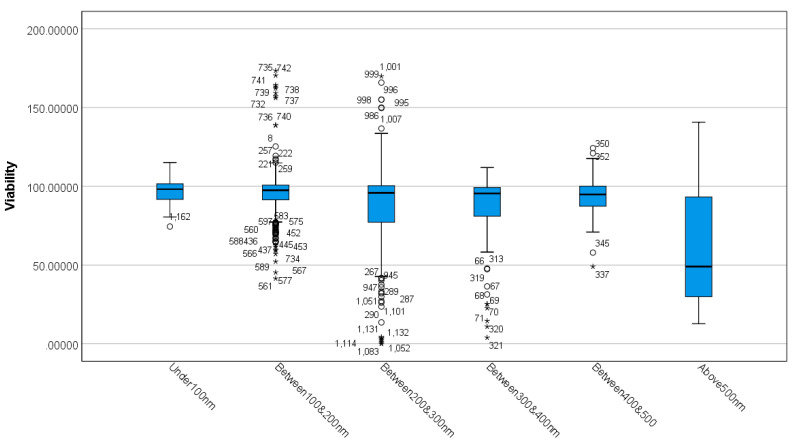
Distribution of viability across different MSN sizes. The boxplot shows the distribution of viability in different size groups. Any data points that are more than 1.5 box-lengths from the edge of their (blue) box are classified by SPSS Statistics as outliers and are illustrated as circular dots. Any data points that are more than 3 box-lengths away from the edge of their box are classified as extreme points (i.e., extreme outliers) and are illustrated with an asterisk (*).

The Shapiro Wilks’s test showed non-normal data for each size group. The boxplot (Figure 3) shows the existence of outliers and extreme outliers in the data.

A Kruskal–Wallis H test was run to determine differences in viability scores between the size groups (Table 2). Distributions of viability scores were not similar for all groups, as assessed by visual inspection of a boxplot (Figure 3). The distribution of viability scores was statistically significantly different between groups, χ^2^ (5) = 75.276, *p* = 0.000.

Subsequently, pairwise comparisons were performed using Dunn’s (1964) procedure with a Bonferroni correction for multiple comparisons. Adjusted *p*-values are presented. Values are mean ranks unless otherwise stated. The post-hoc analysis revealed statistically significant differences in viability scores between above 500 and all the remaining size groups, and “between 300 & 400 nm” (mean rank = 547.81) and “under 100 nm” (mean rank = 710.14) (*p* = 0.013), and “between 200 & 300 nm” (mean rank = 590.52) and “under 100” (mean rank = 710.14) (*p* = 0.018), as seen in Tables S9 and S10 (Supplementary Materials). The boxplot shown in Figure 3, combined with the results of post-hoc analysis, suggests that diameter size affects the toxicity of the MSNs in vitro. Specifically, the results show that MSNs with a diameter size of above 500 nm show lower viabilities and MSNs with a diameter size of under 100 nm showed higher viabilities in vitro.

### 3.2. Differences between Viability Assay Methods Based on Viability

**Figure 4:**
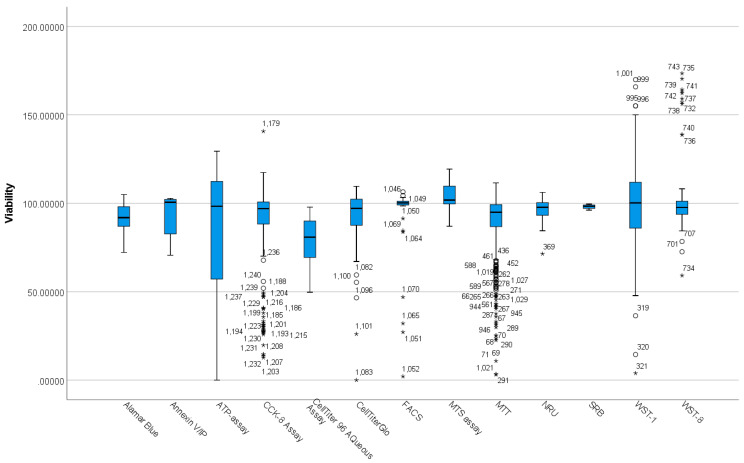
Distribution of viability across different viability assay groups. Data points that are more than 1.5 box-lengths from the edge of their (blue) box are classified by SPSS Statistics as outliers and illustrated as circular dots. Any data points that are more than 3 box-lengths away from the edge of their box are classified as extreme points (i.e., extreme outliers) and are illustrated with an asterisk (*).

The normality test (Shapiro Wilks’s test) shows that viability distributions in functionalisation groups are not normal. The boxplot in Figure 4 shows the existence of outliers and extreme outliers in the data.

As a result, a Kruskal–Wallis H test was run to determine if there were differences in viability scores between the assay groups (Table 3). Distributions of viability scores were not similar for all groups, as assessed by visual inspection of a boxplot. The distribution of viability scores was statistically significantly different between groups, χ^2^ (13) = 81.539, *p* = 0.000. Subsequently, pairwise comparisons were performed using Dunn’s (1964) procedure with a Bonferroni correction for multiple comparisons. Adjusted *p*-values are presented. Values are mean ranks unless otherwise stated.

The post-hoc analysis revealed statistically significant differences in viability scores between CellTiter 96 Aqueous Assay (mean rank = 280.88) and WST-1 (mean rank = 759.74) (*p* = 0.022), CellTiter 96 Aqueous Assay (mean rank = 280.88) and MTS assay (mean rank = 954.15) (*p* = 0.000), CellTiter 96 Aqueous Assay (mean rank = 280.88) and FACS method (mean rank = 770.16) (*p* = 0.043), MTT (mean rank = 557.41) and WST-8 (mean rank = 719.08) (*p* = 0.021), MTT (mean rank = 557.41) and WST-1 (mean rank = 759.74) (*p* = 0.000), MTT (mean rank = 557.41) and MTS assay (mean rank = 954.15) (*p* = 0.000), CCK-8 Assay (mean rank = 631.61) and MTS assay (mean rank = 954.15) (*p* = 0.000), and CellTiterGlo (mean rank = 642.23) and MTS assay (mean rank = 954.15) (*p* = 0.022) as seen in Tables S11 and S12 (Supplementary Materials). The above results and Figure 4 shows that different assay methods produce varied results, for instance, CellTiter 96 AQueous Assay, MTT, and MTS assays produced significantly different results in comparison to other assays.

### 3.3. Differences between Incubation Time Groups on Viability

**Figure 5:**
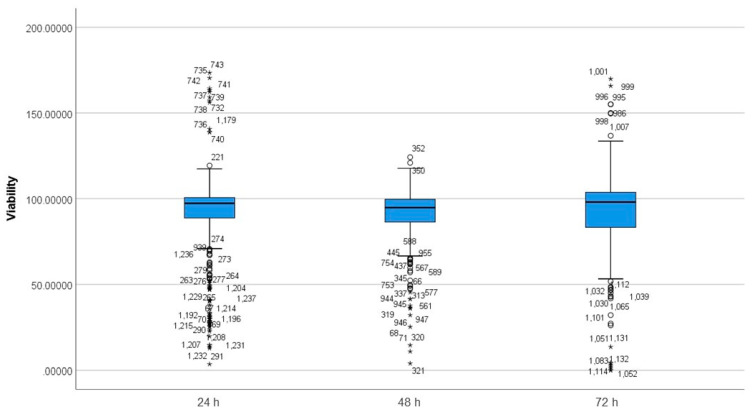
Distribution of viability across different incubation times. The boxplot shows the distribution of viability scores in different incubation durations. Data points that are more than 1.5 box-lengths from the edge of their (blue) box are classified by SPSS Statistics as outliers and illustrated as circular dots. Any data points that are more than three box-lengths away from the edge of their box are classified as extreme points (i.e., extreme outliers) and are illustrated with an asterisk (*).

The Shapiro Wilks’s test demonstrated non-normal distribution in the data, as shown in Figure 5. The boxplot shows the existence of outliers and extreme outliers in the data.

A Kruskal–Wallis H test was run to determine differences in viability scores between the groups that differed in the incubation time (Table 4). Distributions of viability scores were not similar for all groups, as assessed by visual inspection of a boxplot. The distribution of viability scores was significantly different between groups, χ^2^ (2) = 12.007, *p* = 0.002.

Pairwise comparisons were performed using Dunn’s (1964) procedure with a Bonferroni correction for multiple comparisons. Adjusted *p*-values are presented. Values are mean ranks unless otherwise stated. The post-hoc analysis revealed statistically significant differences in viability scores between 48 h (mean rank = 565.56) and 24 h (mean rank = 631.26) (*p* = 0.008), and 48 h (mean rank = 565.56) and 72 h (mean rank = 650.88) (*p* = 0.016) as shown in Tables S13 and S14 (Supplementary Materials). The incubation period is a differentiating factor; the 24 h incubation period is shown to have a smaller range of viabilities and the 72 h incubation period showed the biggest range of viabilities (in accordance with the whisker sizes of the boxplot in Figure 5). The 48 h incubation period’s viability range was bigger than 24 h incubation period and smaller than 72 h incubation period. 

### 3.4. Differences between MSNs’ Zeta Potential Based on Viability

**Figure 6:**
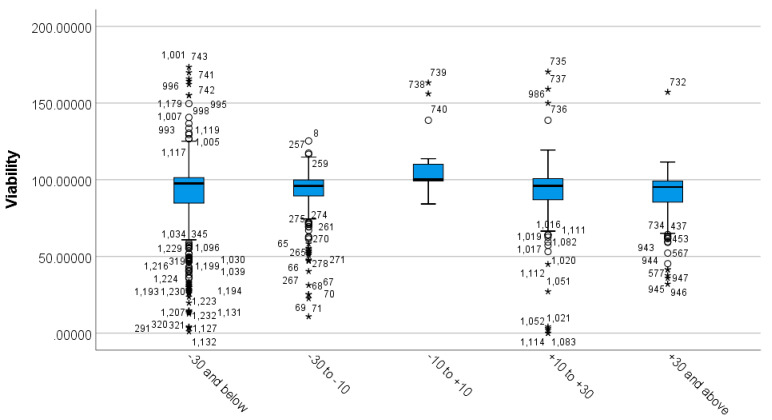
Distribution of viability across different MSN zeta-potential groups. The boxplot shows the distribution of viability across different MSN zeta-potential groups. Any data points that are more than 1.5 box-lengths from the edge of their (blue) box are classified by SPSS Statistics as outliers and are illustrated as circular dots. Any data points that are more than 3 box-lengths away from the edge of their box are classified as extreme points (i.e., extreme outliers) and are illustrated with an asterisk (*).

The Shapiro Wilks’s test showed non-normal distribution of the data. The boxplot (Figure 6) shows the existence of outliers and extreme outliers in the data.

As a result, a Kruskal–Wallis H test was run to determine differences in viability scores between the groups that differed in their zeta-potential value (Table 5). Distributions of viability scores were not similar for all groups, as assessed by visual inspection of the boxplot. The distributions of viability values were statistically significantly different between groups, χ^2^ (4) = 29.115, *p* = 0.000.

Subsequently, pairwise comparisons were performed using Dunn’s (1964) procedure with a Bonferroni correction for multiple comparisons. Adjusted *p*-values are presented. Values are mean ranks unless otherwise stated. The post-hoc analysis revealed statistically significant differences in viability scores between −10 to +10 (mean rank = 939.62) and all other groups (*p* = 0.000), as seen in Tables S15 and S16 (Supplementary Materials). 

The boxplot (Figure 6) shows that zeta potential of lower than −10 mV and higher than +10 mV produces lower viability in vitro, demonstrating that zeta-potential values between −10 mV to +10 mV are associated with highest viability scores.

### 3.5. Differences in Functionalisation Based on Viability

#### 3.5.1. Differences in Surface-Functionalised and Non-Functionalised MSNs Based on Viability

**Figure 7:**
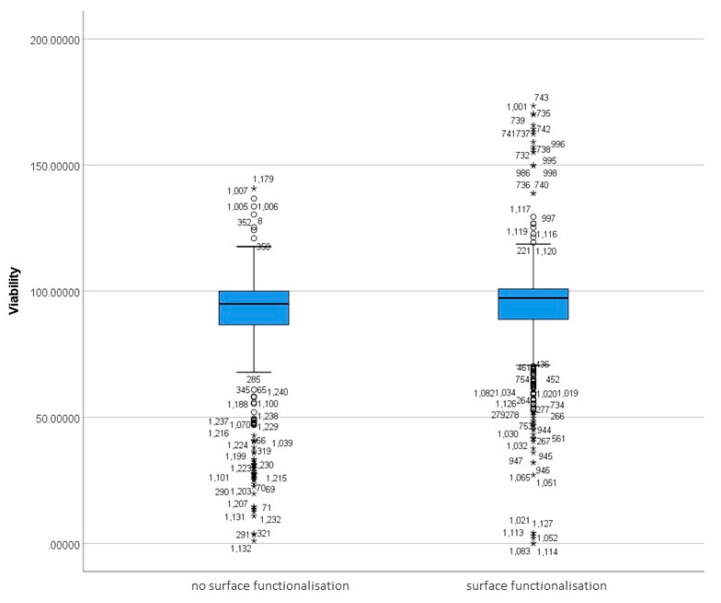
Distribution of functionalised and non-functionalised MSNs viability scores. The boxplot represents the distribution of viability scores in functionalised and non-functionalised MSNs. Any data points that are more than 1.5 box-lengths from the edge of their (blue) box are classified by SPSS Statistics as outliers and are illustrated as circular dots. Any data points that are more than 3 box-lengths away from the edge of their box are classified as extreme points (i.e., extreme outliers) and are illustrated with an asterisk (*).

The distribution of viability measurements was non-normal in both functionalised and non-functionalised MSNs, as assessed by Shapiro–Wilk’s Test, *p* < 0.05. Moreover, the boxplot produced by the SPSS software shows the existence of outliers and extreme outliers in the data (Figure 7).

A Mann–Whitney U test was run to determine differences in viability scores between nanoparticle groups that differed in the presence/absence of surface functionalisation (Table 6). Distributions of the viability scores for surface-functionalised and non-surface-functionalised groups were not similar, as assessed by visual inspection. 

**Figure 8:**
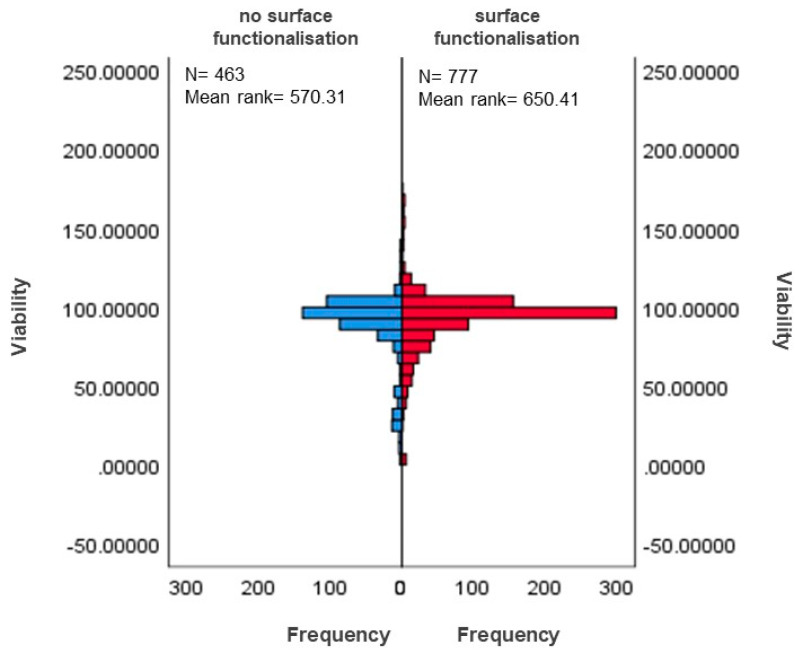
Distributions of functionalised and non-functionalised MSNs viability scores. The graphical representation shows distributions of viability in functionalised and non-functionalised MSNs.

Viability scores for surface-functionalised MSNs (mean rank = 654.41) were statistically significantly higher than for no-surface-functionalisation MSNs (mean rank = 570.31), U = 203113.50, z = 3.810, *p* = 0.000. Figure 7 and Figure 8 show that MSNs with surface functionalisation show a higher viability than MSNs without surface functionalization.

#### 3.5.2. Differences between Functionalisation Groups Based on Viability

**Figure 9:**
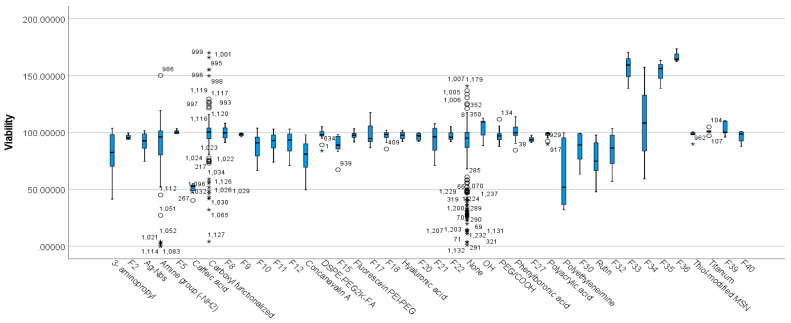
Distribution of viability across different functionalization types. Data points that are more than 1.5 box-lengths from the edge of their (blue) box are classified by SPSS Statistics as outliers and are illustrated as circular dots. Any data points that are more than 3 box-lengths away from the edge of their box are classified as extreme points (i.e., extreme outliers) and are illustrated with an asterisk (*). 4-nitroimidazole-β-cyclodextrin (NI-CD) (F2), amino-functionalised, disulfide bond-bridged (F5), carboxyl groups and Amine-peg2000 and biotinylated rituximab (F8), carboxyphenylboronic acid and sodium alginate (F9), Chlorodimethyloctadecylsilane (F10), Chlorodimethyloctadecylsilane and lipid membrane (F11), Chlorodimethyloctadecylsilane and lipid membrane and Arg-Gly-Asp (F12), fluorescein isothiocyanate (FITC)-labeled (3-aminopropyl)-trimethoxysilane (APTMS) conjugates and polydopamine (F15), Gadolinium and carboxyl group and low-generation peptide dendron (F17), gadolinium-based bovine serum albumin complex (BSA-Gd) and hyaluronic acid (HA) (F18), hydrochloride dopamine (PDA), polyethylene glycol (PEG) (F20), hydrous, and non-crystalline silica (F21), NH2 and Folic acid and arginine-glycine-aspartate (F22), Poly-acrylic Acid and Folic acid (F27), polyethyleneimine-folic acid (F30), succinylated ε-polylysine (F32), tetramethylrhodamine isothiocyanate and aminated (F33), tetramethylrhodamine isothiocyanate and aminated and polyethyleneimine (F34), tetramethylrhodamine isothiocyanate and aminated and polyethyleneimine and polyethyleneglycol (F35), tetramethylrhodamine isothiocyanate and aminated and polyethyleneimine and succinic acid (F36), tumor-specific MUC1 antibody and fluorescein isothiocyanate (F39), upper critical solution temperature (UCST) thermally responsive polymer (F40).

The boxplot produced by the SPSS software shows the existence of outliers and extreme outliers in the data (Figure 9).

A Kruskal–Wallis H test was conducted to determine if there were differences in viability scores between nanoparticle groups that differed in functionalisation types. Distributions of viability scores were not similar for all groups, as assessed by visual inspection of a boxplot.

Viability scores were statistically significantly different between the different functionalisation groups, χ^2^ (39) = 189.917, *p*= 0.000 (Table 7). Subsequently, pairwise comparisons were performed using Dunn’s (1964) procedure. A Bonferroni correction for multiple comparisons was made with statistical significance accepted at the *p* < 0.05 level. Adjusted *p*-values are presented. Values are mean ranks unless otherwise stated.

The post-hoc analysis revealed statistically significant differences in viability scores of three different functionalisation groups, including caffeic acid (mean rank = 78.50), carboxyl-functionalised (mean rank = 791.02), and tumor-specific MUC1 antibody and fluorescein isothiocyanate (F39) (mean rank = 970.68), as seen in Tables S18 and S19 (Supplementary Materials). Figure 9 shows that caffeic-acid-functionalised MSNs produced significantly lower viabilities while carboxyl-functionalised and F39-functionalised MSNs showed significantly higher viability.

### 3.6. Differences between Cell Types Based on Viability

**Figure 10:**
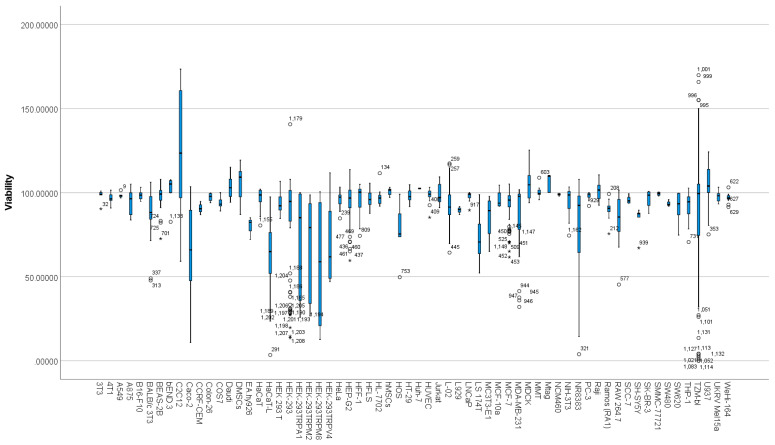
Distribution of viability across different cell types. The boxplot represents the distribution of viability across different cell types. Any data points that are more than 1.5 box-lengths from the edge of their (blue) box are classified by SPSS Statistics as outliers and are illustrated as circular dots. Data points that are more than 3 box-lengths away from the edge of their box are classified as extreme points (i.e., extreme outliers) and are illustrated with an asterisk (*).

Viability scores in cell-type groups showed non-normal distributions based on Shapiro Wilks’s test. Moreover, the boxplot (Figure 10) showed the presence of outliers and extreme outliers in the data.

Consequently, a Kruskal–Wallis H test was run to determine if there were differences in viability scores between the 64 cell types (Table 8). Distributions of viability scores were not similar for all groups, as assessed by visual inspection of a boxplot.

The distributions of viability scores were statistically significantly different between groups, χ^2^ (63) = 279.742, *p* = 0.000. Subsequently, pairwise comparisons were performed using Dunn’s (1964) procedure with a Bonferroni correction for multiple comparisons. Adjusted *p*-values are presented. Values are mean ranks unless otherwise stated. This post-hoc analysis revealed statistically significant differences in viability scores of cell types: HaCaT-L (Mean rank = 161.44), C2C12 (Mean rank = 970.53), Caco-2 (Mean rank = 299.44), U937 (Mean rank = 962.75), LS174T (Mean rank = 211.42), Mtag (Mean rank = 1059.43), DMSCs (Mean rank = 955.45), and Daudi (Mean rank = 956.50), as seen in Tables S20 and S21 (Supplementary Materials). As shown by the post-hoc test and Figure 10, different cells show different viabilities when exposed to MSNs. More specifically, HaCaT-L, Caco-2, LS174T, and DMSCs cell lines showed significantly lower viability scores and C2C12, U937, Mtag, and Daudi cell lines showed significantly higher viability scores.

## 4. Discussion

The application of MSN-based novel drug delivery systems in treating various diseases necessitates assessing their biosafety and biocompatibility. A range of beneficial physicochemical characteristics can influence the success of a therapeutic intervention. These features can control the effective interaction of MSNs at the cellular surface, influence intracellular uptake, and eventually, the payload release/delivery at the intended target site. Paradoxically, the same physicochemical features may exhibit side effects, govern cell-specific toxicity as a function of time, concentration, intra/extracellular environment, and spatiotemporal localisation. 

To better understand the toxicity potential of MSNs and to provide a coherent analysis of the underlying toxicity parameters, data from 45 papers were collected, analysed to obtain the mean ranks of the study groups, and a post-hoc test was carried out by IBM SPSS Statistics 26 (Table 1, Tables S1 and S2). This study assessed the relationship between critical parameters, including surface functionalisation, zeta potential and particle size of MSNs, and cytotoxicity, by investigating differences among groups. Indeed, these parameters play an essential role in agglomeration of MSNs in biological media, protein corona formation, interactions with cellular receptors, intracellular trafficking, and cytotoxicity [63,64]. Additional factors, such as experimental conditions, may also have negative influences on cell viability. Experimental conditions, such as incubation time, cell types used in various research studies, and the compatibility of different cytotoxicity assays, were, therefore, included in the analysis as crucial parameters in risk assessment and predicting the behaviour of MSNs. 

Diameter size: This study assessed the cell viability of MSNs as a function of size, which ranged from <100 nm to >500 nm. Out of the 45 studies, 596 experimental cases had an MSN size ranging from 100 nm to 200 nm; 309 cases used MSN particles ranging from 200 nm to 300 nm, as seen in Table S9 (Supplementary Materials). The most significant differences were observed with particle sizes below 100 nm and above 500 nm (Figure 3, Table 2 and Table S10). This is expected as larger particle size may not be suitable for effective cellular uptake and may have contributed to limited cytotoxicity. A study by Yang et al. (2019) showed that an optimal DLS particle size of 98.35 nm was effective for the selective uptake of MSNs by leukaemia cells in culture [32]. The correlation between cytotoxicity and size has been determined by many studies [65,66,67,68]. In-vitro experiments have shown higher cytotoxicity of nanoparticles compared to their corresponding microparticles [67,69,70,71,72]. 

Indeed, smaller particles (<100 nm) can potentially induce relatively high toxicity and show more cytotoxic and inflammatory potency. Size-dependent cytotoxicity has been widely perceived and well documented as small particles have a larger surface area to produce greater toxicity example, by inducing higher oxidative stress [73,74,75]. The surface area has been shown to play a pivotal role in enhancing surface reactivity. 

Studies have shown that although nonphagocytic cells show a positive correlation between small size and increased cytotoxicity, macrophages and monocytes showed a more cytotoxic response, subsequent to exposure with microparticles vs. nanoparticles. For example, no cell damage in THP-1 cells was seen after exposure to 30–70 nm silica NPs, compared to cytotoxicity induced by 1000 nm particles [76]. In contrast to the above studies, no significant differences in toxicity have been reported for 10–100 nm silica particles compared to 45 μm when tested in many other cell lines [77,78]. 

Size-dependent agglomeration of these nanoparticles may also play a critical role in determining cytotoxicity. A study has shown comparable cytotoxicity results with 15 nm and 46 nm silica particles due to agglomeration. The DLS data showed agglomeration of 15 nm to form hydrodynamic sizes of 590 nm, and the 46 nm silica nanoparticles agglomerated to 617 nm. Therefore, the similar cytotoxicity in the different-sized particles can be explained by similar hydrodynamic sizes [79]. However, another study demonstrated that although smaller MSN particles of 12 nm and 25 nm can result in higher agglomeration resulting in comparable hydrodynamic diameters to their larger-sized counterparts (600 nm), as indicated by the DLS measurement, the smaller particles still showed more potent toxicity [63]. 

Contrarily, a study on human red blood cell–MSN interaction showed that small MSNs of ~100 nm size adsorbed to the surface membrane of the red blood cells without altering the surface of the cells or their morphology, in contrast to MSNs of ~600 nm that adsorbed on the cell’s surface and induced membrane deformation and potential haemolysis [80]. Similarly, a study on HepG2 and THP-1 cells demonstrated that larger MSN particles (>100 nm) induced more cytotoxicity [81]. Another study showed similar results where larger MSNs (≥100 nm) at higher treatment doses (≥500 μgmL^−1^) resulted in a necrotic cell death that correlated with increased cellular accumulation of MSNs, a significant increase in oxidative stress, and NF-κB and AP-1-mediated inflammatory gene upregulation [50].

Generally, smaller particle size associated with higher surface area has a potentially larger reactive surface to induce toxicity. Interestingly, even though MSNs have a higher surface area compared to their size equivalent nonporous silica, they generally show lesser cytotoxic potency as measured by their haemolytic activity. This is because other parameters, such as shape, surface charge, and porosity, may modulate uptake and, hence, the toxicity profile of MSNs. This highlights the need for a case-by-case evaluation of silica particles, and an assumption of higher surface related to small size should be considered with caution. However, there are several reports in the literature that show a positive correlation between toxicity and particle size [63,67,69,70,71,72]. Therefore, it is apparent that a correlation of physicochemical features of MSNs to their toxicological response is complex and requires an in-depth testing strategy that can evaluate the various factors that could potentially contribute to the biological response generated by MSNs under different experimental conditions. 

Viability assay: An appropriate viability assay must be chosen to measure the toxicity of the test nanoparticle. This is to avoid false positives or false negatives due to the interference with the MSNs, which themselves may lead to misinterpretation of the results [82,83]. 

There are many different types of cytotoxicity assays, such as Alamar Blue, CCK-8 Assay, CellTiterGlo Assay, MTS Assay, MTT and WST-1, as seen in the data collected and analysed in this study (Figure 4). The most commonly used assay is MTT (N = 648), followed by CCK-8 assay (N = 246) and WST-1 (N = 86), as seen in Table S11 (Supplementary Materials). The boxplot (Figure 4; Table 3) shows several outliers, mainly for CellTiterGlo, MTT, MTS, WST-1, and WST-8. Most of the significant difference in the pairwise comparison is due to the spread of the values and the outliers, as seen in Figure 4; Table S12 (Supplementary Materials). Further, significant differences in the N values may not allow reasonable interpretation of the pairwise comparisons. However, there seem to be outliers towards higher cell viability detected with WST-1 and WST-8 assays, while more outliers towards the lower cell viability end for MTT and CCK-8 assay. It has been observed that endocytosis of mesoporous silica nanoparticles (MSNs) is linked to the exocytosis of formazan crystals and may, therefore, interfere with the MTT assay. This has been shown in HeLa cells and astrocytes [84]. The perturbed intracellular vesicle trafficking subsequent to MSN uptake seems to be related to exocytosis of formazan and overestimates the cytotoxicity of mesoporous silica nanoparticles compared to other cytotoxicity tests, such as WST-1 test and flow cytometry. Although the overall spread of data follows a similar pattern for MTT and FACS in the present study, the latter may not accurately represent MSN-induced changes in cell viability due to the small N number. While MTT points towards overestimation of cytotoxicity related to MSNs, WST-1, on the other hand, was found to underestimate cytotoxicity and exceed 100% viability, as reflected in this study. These inaccuracies seem to be based on background responses that positively correlated with MSN concentration when tested under acellular conditions. This has also been attributed to increased cell proliferation kinetics induced by MSNs [60]. 

Most routinely used assays, including MTT and WST-1, are tetrazolium-based assays that rely on redox processes related to cell metabolism for the read-out, and these assays have been shown to interfere with other particles [79,83,84,85]. Therefore, to obtain reliable and reproducible data sets for cytotoxicity induced by MSNs, other assays based on ATP activity, FACS, and cell count need to be considered. Different assays may give varied results for the same MSNs depending on various functional groups or other parameters, which may affect the assay. Therefore, more than one assay is recommended to assess the toxicity potential of various MSNs to obtain an accurate evaluation of their toxicity profile.

Exposure/incubation time: An important parameter in any toxicological evaluation is the duration of exposure, which, in most studies, is 24 h, as reflected by the data analysis in the present study of 567 studies conducted at 24 h of exposure to MSNs, as seen in Figure 5. This study also included studies conducted at 48 h (N = 467) and 72 h (N = 183), as seen in Table S13 (Supplementary Materials), and included significant differences between the three time points, as shown in Table 4 and Table S14 (Supplementary Materials). Although other studies have been conducted ranging from 2 h to 12 h, these may not be sufficiently relevant to any toxicological assessment as contrary to chemical diffusion, movement, and uptake of nanomaterials into cells is slower and can take as long as 24 h or more. Multi-endpoint toxicological assessment subsequent to ≥24 h exposure to MSNs or any other nanomaterial is, therefore, more realistic and necessary as toxicity may take longer to develop. Correlation between exposure time and toxicity has been seen in many studies. A study by Yazdimamaghani et al. (2019) showed a time-dependent (ranging from 4 h to 24 h) toxicity profile of nonporous silica and MSNs, with the most observed toxicity at 24 h. They also showed a direct correlation between increased time of exposure to MSNs and the number of genes that showed alteration in gene expression [86]. 

Another study evaluated the toxicity of silica nanoparticles on A549 human lung cancer cells. It was found that the cytotoxicity of these nanoparticles increased in a time-dependent manner. Out of the three time points studied at 24 h, 48 h, and 72 h, the lowest cell viability was seen at 72 h. The results indicate that increased cytotoxicity is due to oxidative stress and from the likely penetration of nanoparticles into the cell nucleus over a long period, resulting in aberrant clusters of topoisomerase I and protein aggregation that can cause perturbation of various processes, including replication, transcription, and cell proliferation [79].

A study assessing the cytotoxicity of amorphous silica nanoparticles on L-02 hepatocyte cells showed that a longer exposure time of nanoparticles on the cells could increase toxicity due to the kinetic accumulation of cell damage. The longer exposure time can increase the cellular uptake of the nanoparticles; therefore, as the time increases, the cell viability decreases [87].

Although many studies investigate exposure effects of MSNs at 24 h or more, it is essential to note that at the cellular level, earlier time points (4 h) could be necessary as early-stage lysosomal pathway activation by the MSNs may result in lysosomal overload. This could influence gene expression responses at the transcriptional level of vacuolar H+(V)-ATPase genes involved in proton transport. These genes regulate lysosome acidification with the help of membrane and peripherally associated protein complexes, potentially disrupting their association and, hence, their ATPase activity [88]. The study concluded that MSN could induce early lysosome alkalisation by influencing membrane and peripherally associated protein complexes of vacuolar H+(V)-ATPase expression levels. This could significantly impact the endo-lysosomal trafficking of MSNs and the associated payload [88]. Therefore, studies encompassing a wide range of time points would be more informative if detailed toxicological assessments were carried out on potential MSN–drug entities. 

Zeta potential: Studies have shown that positively charged (cationic) nanoparticles can cause membrane damage. Positively charged nanoparticles have a higher cellular uptake when compared to negatively charged particles [89]. A correlation was observed between cationic surface charge and significant immune response, as well as an increase in cytotoxicity in nonphagocytic cells compared to neutral or anionic particles [64]. However, in phagocytic cells, anionic MSNs are more cytotoxic and can cause intracellular damage as MSNs with a negative zeta-potential value can undergo opsonisation, enhancing their interaction with macrophage receptors, leading to their rapid phagocytosis by macrophage [78].

The present study showed that the cell viability differences were significant between the zeta-potential values of −10 to +10 mV (N = 29 as shown in Table S15) compared to all other zeta-potential groupings, including −30 to −10 mV, +10 to +30 mV, and +30 and above mV, as shown in Table S16 (Supplementary Materials). The studies that showed the zeta potential of MSNs between −10 to +10 mV showed less toxicity than all the other groups (Figure 6, Table 5). Varied amounts of endocytosed particles depending on the zeta-potential values have been noticed in C2C12 myoblasts. The order of endocytosis was MSN-PEI-PEG < MSN-NH2 < MSN-PEI < MSN-PEI-SUCC, with the corresponding Z-potential values of +4.2, +24.6, +35.3, and −31.8, respectively. The endocytosis and Z potential correlated with significant toxicity observed after exposure to MSN-PEI and MSN-PEI-SUCC. On the other hand, a low surface charge may lead to low cellular uptake, which is determined by the low interaction and affinity towards the negatively charged cell membrane [51]. 

The study also showed the most agglomeration for MSN-PEI-PEG and least for MSN-PEI-SUCC. Higher zeta potential may prevent agglomeration, increasing the cell surface affinity of MSNs and consequently their cellular uptake, resulting in a more cytotoxic response. Therefore, it is crucial to understand that the differential zeta-potential values may determine cytotoxicity by influencing the resultant agglomeration, which may be a decisive factor that governs the uptake of MSNs and the subsequent effect on the viability of cells. 

Functionalisation: The two functional surfaces of MSNs, comprising the cylindrical pore surface and exterior particle surface, contain the silanol groups [90]. The external surface can be conjugated with targeting ligands, which facilitate efficient cell-specific drug delivery [91]. Many functional moieties can be attached to the surface of MSNs via covalent conjugation/interactions with surface silanol groups, which are formed during the fabrication of MSNs. This allows better control over drug loading and its subsequent release at the precise target. The functional groups include various molecules, such as carboxylate, amine, polyethylene glycol, and carboxylate groups. Functional groups may negatively interact with constituents of the cell membrane and impair the structure and function of these molecules. Therefore, biocompatible surface modification is an essential step in controlling undesirable surface reactivity while enhancing the biomedical applicability of MSNs [64,84,92]. 

The present study also showed statistically significant differences in cell viability between surface-functionalised MSNs and non-functionalised MSNs (Figure 7 and Figure 8). Although the statistical significance observed in the present study indicates that functionalisation may confer a protective effect on cell viability, the current results cannot be generalised or extrapolated. This is because the study comprised unequal sample sizes, N = 777 for the functionalised MSN vs. N = 463 for non-functionalised MSN (Figure 8; Table 6). The normality test (Shapiro Wilks’s test) shows that viability distributions in functionalisation groups are not normal, as seen in Table S17 (Supplementary Materials).

The study showed statistically significant differences in viability scores of three different functionalisation groups, which included caffeic acid (N = 8), carboxyl functionalised (N = 138), and F39 (N = 14) (Figure 9, Table 7) (Tables S18 and S19; Supplementary Materials). All these studies were performed under different experimental conditions with varied physicochemical features, with N ranging from 4 to 138; thus, establishing apparent differences in cell viability based on the functionalisation groups mentioned above is difficult. 

However, it is interesting to note that a significant number of studies have fabricated MSNs with biocompatible yet effective functionalisation groups. Indeed, this is important, along with other optimised physicochemical characteristics, to achieve maximal efficacy in safely targeting and delivering the therapeutic load into the tumour tissue. Of particular importance is the need for the endo-lysosomal escape of the MSN–drug entity into the cytoplasm or nucleus, which is crucial to circumvent lysosomal degradation and enhance the drug load’s effectiveness to intracellular targets. This can be achieved by attaching a cationic functional group in the acidic endo-lysosomal compartment, causing its interaction with the endo-lysosomal membrane and escaping into the cytosol [93]. 

Nevertheless, there are reports of enhanced mesoporous silica degradation, even in the intracellular or the extracellular milieu. Therefore, it becomes all the more important to (1) understand the spatiotemporal escape of MSNs (based on their functional group) from the lysosomal compartments, (2) evaluate the conditions that result in their breakdown, and (3) assess the changes that may occur with the payload until they reach the desired therapeutic target site [93,94]. 

Functionalisation groups are also important to target certain specific cell receptors, e.g., folic acid, that can substantially improve the recognition and cellular uptake of MSNs [95]. Therefore, surface modification of NPs represents an essential strategy to successfully develop specific and biocompatible nano-platforms for precise and sensitive therapy and diagnosis. It is important to highlight here that surface functionalisation is a critical determinant that affects cellular toxicity by modulating the uptake of MSNs. Although higher uptake may correlate with a significant decrease in cell viability, penetration through biological membranes is a desirable feature; it is a prerequisite for efficient drug delivery. It provides the option to lower the amount of dose delivered [51,81].

Cell types: The analysis in the present study showed that the cell lines most studied included HepG2 (N = 99), HEK293 (N = 97), MCF-7 (N = 100), and TZM-bl (N = 155), representing liver, kidney, epithelial breast cancer, and a clone of HeLa cells, respectively (Table S20; Supplementary Materials). These represent tissues that are either the site of metabolism and excretion (HepG2 and HEK293) of MSN-loaded drugs or cell lines that are ubiquitously used as in-vitro models in cancer research (MCF-7 and TZM-BL). As seen in Table S21 (Supplementary Materials), the post-hoc analysis revealed statistically significant differences in viability scores of studies that used LS174T and Caco-2 cells (both include colon adenocarcinoma cells), C2C12 human myoblasts muscle cells, HaCaT-L keratinocyte cell line, Daudi, U937 myeloid leukaemia cell line, and DMSCs human Decidua-derived mesenchymal stem cells.

Exposure to nanomaterials and, in the present study, MSNs has been demonstrated to cause cell-type-dependent changes in cell viability. This can be attributed to factors, such as differential sensitivity of specific cell types, p53 competency, and varied cellular uptake mechanisms [63,96]. Different uptake levels and intracellular accumulation can affect cytotoxicity, e.g., cellular uptake of silica nanoparticles in THP-1 macrophages and A549 cells has been observed to correspond with more substantial toxicity, as compared to HaCaT and NRK52E cells, which showed less cellular uptake [97]. Interestingly, another study showed the extent of cellular uptake of NPs to be similar in all the three cell lines (BT-474, SCC7, and SH-SY5Y) under investigation [98,99]. 

Cell-type-specific membrane-bound receptors influence the uptake and cytotoxicity of MSNs into various cellular compartments by specifically interacting and binding the functionalised or non-functionalised MSNs. Cells that lack receptors for specific nanoparticles can easily be internalised by adsorptive endocytosis or fluid-phase pinocytosis [91,94]. Differential expression levels of integrin αvβ3 on the surface of the cells have been seen to facilitate specifically functionalised MSNs to enter cells via α_v_β_3_-integrin receptor-mediated endocytosis in various cell lines, including A549, MCF-7, and HEK293 T [100,101,102,103,104]. 

Endocytosis, either clathrin-mediated or caveolin-mediated, is the most common mechanism of uptake of silica nanoparticles, as described by studies on various cell lines, including, HepG2 [97,105], THP-1 [106], A549 [106,107], and HeLa [108], and parameters (most importantly size range of the nanoparticles) govern the mechanism that is involved in mediating the uptake of these particles [105,108,109,110]. 

Although several physicochemical characteristics may have contributed to the toxicity differences observed in this study, it is essential to understand other inherent features characteristic of cell types that may influence the resultant toxicity after MSN exposure. For instance, C2C12 cells that show significantly increased differences than the overall mean have been associated with showing resistance to toxicity at a high passage number of >60. This has been attributed to the depletion of mitochondrial DNA and alterations in the function of Bad, Bax, and caspase-3 [111]. Therefore, information on passage numbers and limiting experiments to specific passage numbers are instrumental in avoiding discrepancies in data collection when performing assessments based on statistical analysis (Figure 10; Table 8). 

Another intrinsic feature was observed in U937 monocytes. This cell line showed an increased cell proliferation related to immunological response subsequent to MSN internalisation [39]. Our analysis has shown similar results; a higher median toward increased cell viability is observed, as shown in Figure 10. Reports on mitochondrial enzyme activation, the proliferation of monocytes to external stimuli, hormesis, and monocytosis have been reported to explain the increased cell viability [112,113]. Interestingly, the study used a WST-1 assay to measure cell viability, which may be associated with the confounding effects of MSN exposure on cytotoxicity assays/results, as indicated in the present analysis [39]. 

Furthermore, it is of paramount importance to identify cell lines for p53 competency, as a genetically unstable cell line with an impaired p53 could compromise genome integrity and lead to false-positive or misleading results. Some of the cell lines analysed in this study have shown significant differences in cytotoxicity compared to the overall cell viability results. Interestingly, a decrease or increase in cell viability in those cell types after MSN exposure did not show any correlation with either the presence of p53 mutation in those cell lines (HaCaT-L, Caco-2, U937, DMSCs and Daudi cells) or the absence (C2C12, LS174T) [96,114,115,116,117,118,119,120], highlighting the role of other features in influencing the cytotoxicity results. 

Therefore, choosing a cell line relevant to the specific application of a given MSN (or including payload) being tested is essential to understand the mechanism of cellular uptake of MSNs for future development of new strategies and efficient drug delivery. Cellular uptake (including entry into mitochondria or nucleus) and its consequence on the cytotoxic potency or inflammatory potential is also cell type-specific [63].

## 5. Conclusions

Understanding and evaluating the risk assessment of MSNs is important, as detailed in Table 9. Although MSNs are generally considered biocompatible, many studies have assessed their toxicity and have demonstrated their potential to cause adverse effects. 

The overall results demonstrate functionalisation-dependent changes in cell viability. Therefore, toxicological assessment of functionalised vs. non-functionalised MSNs (for a given MSN under study) needs to be included to identify the cause of toxicity and guide the fabrication of biocompatible MSNs. Although it is well established that this could influence the zeta potential, agglomeration of MSNs, cellular uptake, and downstream toxicity, an additional layer of protein corona (determined by the proteins in the biological media) can alter cell viability via changes in the physicochemical characteristics and their surface reactivity. Many key cellular effects and processes, such as MSN targeting, cellular uptake, and intracellular trafficking, are dictated by cell surface receptors’ initial recognition of MSNs [121]. Therefore, determining constituents of the protein corona may help identify the plausible interaction of MSNs with the cell surface. However, this requires understanding the target cell type as the expression of various receptors specific to cell types is different. This may allow one to predict the corona–cell receptor interactions and aid in the discovery of novel receptors that can be exploited for targeting MSN-based nano-carriers [122]. Moreover, pores in the MSN may allow adsorption of proteins in the protein corona in exchange for drug payload. Therefore, the experimental conditions comprising the type and constitution of media need to be considered. A thorough in-vitro investigation needs to be carried out to minimise premature loading off [123].

**Figure 11:**
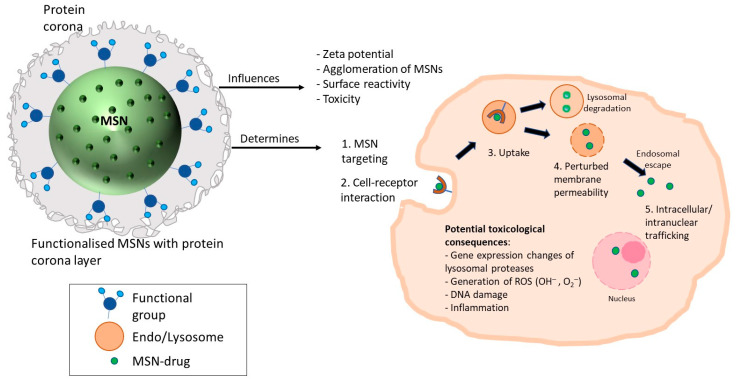
Protein corona can influence physicochemical characteristics of MSNs. This may determine MSN targeting, receptor recognition, uptake, intracellular and/or intranuclear trafficking, and potential toxicological consequences.

As mentioned before, functionalisation-dependent trafficking of internalised MSNs, intracellularly or intercellularly [124], warrants case-by-case investigation, as the final payload delivery at the intended target site is of paramount importance for effective therapy. Additionally, time-dependent lysosomal uptake and its dysfunction, including gene expression of lysosomal proteases, perturbation of membrane permeability, or disruption of its internal structures, could have toxicological consequences and should be considered when assessing the impact of MSN exposure (Figure 11). A range of time points should be considered as these may bring about a subtle cellular alteration in the cellular components, such as DNA damage and other modifications in gene expression. 

A variety of critical factors influence the toxicity assessment of MSNs, and the scientific community needs to aim for a detailed toxicology assessment in the future, as well as a consistent approach that is critical in developing and supporting the progression of MSNs as promising vehicles in various applications.

## Figures and Tables

**Table 1:** Shapiro Wilk’s Test of Normality for each study in the dataset.

Reference	Studies	Statistic	df	Sig.
[19]	Wright et al., 2021	0.928	6	0.565
[20]	Zhao et al., 2019	0.895	18	0.047
[21]	Wu et al., 2019	0.95	12	0.632
[22]	Yan et al., 2020	0.911	16	0.122
[23]	Kundu et al., 2020	0.95	6	0.74
[24]	Deng et al., 2021	0.933	12	0.415
[25]	Kim et al., 2021	0.952	30	0.188
[26]	Vejdani Noghreiyan et al., 2020	0.967	5	0.854
[27]	Zhang et al., 2020	0.948	36	0.089
[28]	Ali et al., 2020	0.873	6	0.238
[29]	Wang et al., 2019	0.883	12	0.096
[30]	Khatoon et al., 2018	0.891	5	0.364
[31]	Maksimović-Ivanić et al., 2019	0.992	9	0.998
[32]	Yang et al., 2019	0.975	32	0.648
[33]	Xie et al., 2016	0.885	10	0.151
[34]	Paris et al., 2016	0.902	10	0.228
[35]	Guo et al., 2016	0.945	32	0.103
[36]	Ebabe Elle et al., 2016	0.974	32	0.63
[37]	You et al., 2016	0.952	6	0.754
[38]	Zhang et al., 2016	0.945	8	0.664
[39]	Mannerström et al., 2016	0.842	64	0
[40]	T. Li et al., 2017	0.916	6	0.48
[41]	Ferrauto et al., 2017	0.897	6	0.358
[42]	Chen et al., 2016	0.911	28	0.021
[43]	Cheng et al., 2017	0.955	20	0.441
[44]	Fei et al., 2017	0.931	96	0
[45]	Zhou et al., 2017	0.973	30	0.638
[46]	Nguyen et al., 2017	0.932	44	0.012
[47]	Dréau et al., 2016	0.816	14	0.008
[48]	Kienzle et al., 2017	0.958	17	0.599
[49]	Liu et al., 2017	0.873	29	0.002
[50]	Chou et al., 2017	0.905	72	0
[51]	Paatero et al., 2017	0.75	12	0.003
[52]	Y. Li et al., 2017	0.899	6	0.371
[53]	Martínez-Carmona et al., 2018	0.95	8	0.713
[54]	Hei et al., 2017	0.825	4	0.155
[55]	Gounani et al., 2018	0.865	144	0
[56]	Saroj and Rajput, 2018	0.758	24	0
[57]	Tran et al., 2018	0.863	10	0.084
[58]	Li et al., 2018	0.687	32	0
[59]	Hou et al., 2018	0.96	6	0.816
[60]	Braun et al., 2018	0.913	155	0
[61]	Guo et al., 2018	0.913	16	0.129
[62]	Lu et al., 2018	0.788	28	0
[15]	Mohammadpour et al., 2019	0.902	64	0

**Table 2:** Independent-Samples Kruskal–Wallis H Test Summary. The table shows the independent samples Kruskal–Wallis H test between MSN diameter size groups’ viability scores.

Total N	1240
Test Statistic	75.276 ^a^
Degree Of Freedom	5
Asymptotic Sig.(2-sided test)	0.000

^a^ The test statistic is adjusted for ties.

**Table 3:** Independent-Samples Kruskal–Wallis H Test Summary. The table shows independent samples Kruskal–Wallis H test summary between viability assay method groups’ viability scores.

Total N	1236
Test Statistic	81.539 ^a^
Degree Of Freedom	12
Asymptotic Sig.(2-sided test)	0.000

^a^ The test statistic is adjusted for ties.

**Table 4:** Independent-Samples Kruskal–Wallis H Test Summary. The table shows the independent samples Kruskal–Wallis H test between various incubation time groups’ viability scores. *p*-value under 0.05 indicate significant differences.

Total N	1217
Test Statistic	12.007 ^a^
Degree Of Freedom	2
Asymptotic Sig. (2-sided test)	0.002

^a^ The test statistic is adjusted for ties.

**Table 5:** Independent-Samples Kruskal–Wallis H Test Summary. The table shows the results of the independent-samples Kruskal–Wallis H test summary between MSN zeta-potential groups’ viability scores.

Total N	1240
Test Statistic	29.115 ^a^
Degree Of Freedom	4
Asymptotic Sig. (2-sided test)	0.000

^a^ The test statistic is adjusted for ties.

**Table 6:** Independent-Samples Mann–Whitney U Test between functionalised and non-functionalised MSNs. The table shows the independent-samples Mann–Whitney U test results between functionalised and non-functionalised MSNs’ viability scores. *p* value lower than 0.05 indicates significant differences.

Total N	1240
Mann–Whitney U	203,113.500
Wilcoxon W	505,366.500
Test Statistic	203,113.500
Standard Error	6099.500
Standardised Test Statistic	3.810
Asymptotic Sig.(2-sided test)	0.000

**Table 7:** Independent-Samples Kruskal–Wallis H Test Summary. The table shows the results of independent-samples Kruskal–Wallis H test between functionalisation groups’ viability scores.

Total N	1239
Test Statistic	189.917 ^a^
Degree Of Freedom	39
Asymptotic Sig.(2-sided test)	0.000

^a^ The test statistic is adjusted for ties.

**Table 8:** Independent-Samples Kruskal–Wallis H Test Summary. The table shows the independent-samples Kruskal–Wallis H test between cell type groups’ viability scores. *p*-values under 0.05 indicate significant differences.

Total N	1240
Test Statistic	279.742 ^a^
Degree Of Freedom	63
Asymptotic Sig.(2-sided test)	0.000

^a^ The test statistic is adjusted for ties.

**Table 9:** Summary of the various parameters assessed in this study and considerations that may influence cytotoxicity results.

Parameters	Considerations
Diameter Size	Size-dependent differential agglomeration
Exposure/incubation time	Time-dependent decrease in cell viability due to increased cellular uptake or intracellular accumulation; Shorter incubation time could cause lysosomal pathway activation by the MSNs
Zeta Potential	Low zeta potential values may correlate with reduced cellular uptake and decreased cytotoxicity
Functionalisation	Biocompatible surface modification to control undesirable surface reactivity; Along with an additional layer of protein corona, influences zeta potential, agglomeration of MSNs, cellular uptake, and downstream toxicity; Functional groups that allow endo-lysosomal escape (e.g., cationic in the acidic endo-lysosomal compartment) of MSN-drug entity into the cytoplasm or nucleus to enhance the efficacy of the drug load to intracellular targets; Functional groups that target specific cell receptors to ensure precise and sensitive therapy and/or diagnosis
Viability assay	Testing under acellular conditions; Employ multiple assays for cytotoxicity assessments
Cell type	Differential sensitivity of specific cell types; Varied cellular uptake mechanisms e.g., clathrin-mediated or caveolin-mediated endocytosis that result in different levels of uptake and intracellular accumulation; Cell type-specific receptor-mediated selective uptake; Identify cell lines for p53 competency

## Data Availability

Data is contained within the article or supplementary material.

## Supplementary Materials

The following are available online at https://www.mdpi.com/article/10.3390/nano12122016/s1: Table S1. Mean ranks of study groups; Table S2. Post-hoc test of study groups; Table S3. Normality tests for different MSN sizes; Table S4. Normality tests for different viability assay method groups; Table S5. Normality tests for different incubation times; Table S6. Normality tests for different MSN zeta-potential; Table S7. Normality tests for different functionalisation types; Table S8. Normality test for cell types’ viability; Table S9. Mean ranks of MSN size groups; Table S10. Post-hoc test of MSN size groups; Table S11. Mean ranks of viability assay method groups; Table S12. Post-hoc test of viability assay method groups; Table S13. Mean ranks of incubation time groups; Table S14. Post-hoc test of incubation time groups; Table S15. Mean ranks of MSN zeta-potential groups; Table S16. Post-hoc test of MSNs’ zeta-potential groups; Table S17. Shapiro Wilk’s test of normality for functionalised and non-functionalised MSN groups in the dataset; Table S18. Mean ranks of functionalisation groups; Table S19. Post-hoc test of functionalisation groups; Table S20. Mean ranks of cell-type groups; Table S21. Post-hoc test of cell type groups.
