# Supplementary material for: Influence of Critical Parameters on Cytotoxicity Induced by Mesoporous Silica Nanoparticles
Source: Nanomaterials (Basel). 2022 Jun 11;12(12):2016. doi: 10.3390/nano12122016 (PMC9228019; doi:10.3390/nano12122016)
Supplement: Supplementary file 1 [file nanomaterials-12-02016-s001.zip › nanomaterials-1685555-supplementary.pdf]

**Supplementary Materials**

**Table S1. Mean ranks of Study groups.**

| | Study Groups | N | Mean Rank |
|---|---|---|---|
| Viability | Wright et al., 2021 | 6 | 958.17 |
| | Zhao et al., 2019 | 18 | 756.25 |
| | Wu et al., 2019 | 12 | 828.17 |
| | Yan et al., 2020 | 16 | 678.44 |
| | Kundu et al., 2020 | 6 | 498.58 |
| | Deng et al., 2021 | 12 | 134.00 |
| | Kim et al., 2021 | 30 | 681.28 |
| | Vejdani Noghreiyan et al., 2020 | 5 | 918.50 |
| | Zhang et al., 2020 | 36 | 687.26 |
| | Ali et al., 2020 | 6 | 602.42 |
| | Wang et al., 2019 | 12 | 879.71 |
| | Khatoon et al., 2018 | 5 | 603.80 |
| | Maksimović-Ivanić et al., 2019 | 9 | 453.78 |
| | Yang et al., 2019 | 32 | 403.19 |
| | Xie et al., 2016 | 10 | 483.90 |
| | Paris et al., 2016 | 10 | 953.75 |
| | Guo et al., 2016 | 32 | 731.28 |
| | Ebabe Elle et al., 2016 | 32 | 142.03 |
| | You et al., 2016 | 6 | 334.00 |
| | Zhang et al., 2016 | 8 | 616.13 |
| | Mannerström et al., 2016 | 64 | 587.49 |
| | T. Li et al., 2017 | 6 | 699.33 |
| | Ferrauto et al., 2017 | 6 | 925.42 |
| | Chen et al., 2016 | 28 | 700.57 |
| | Cheng et al., 2017 | 20 | 533.63 |
| | Fei et al., 2017 | 96 | 447.24 |
| | Zhou et al., 2017 | 30 | 879.18 |

| | | | |
|---|---|---|---|
| | Nguyen et al., 2017 | 44 | 417.00 |
| | Dréau et al., 2016 | 14 | 969.68 |
| | Kienzle et al., 2017 | 17 | 692.68 |
| | Liu et al., 2017 | 29 | 761.76 |
| | Chou et al., 2017 | 72 | 647.11 |
| | Paatero et al., 2017 | 12 | 1130.75 |
| | Y. Li et al., 2017 | 6 | 642.33 |
| | Martínez-Carmona et al., 2018 | 8 | 280.75 |
| | Hei et al., 2017 | 4 | 699.88 |
| | Gounani et al., 2018 | 144 | 750.61 |
| | Saroj and Rajput, 2018 | 24 | 734.58 |
| | Tran et al., 2018 | 10 | 407.70 |
| | Li et al., 2018 | 32 | 566.77 |
| | Hou et al., 2018 | 6 | 730.00 |
| | Braun et al., 2018 | 155 | 682.31 |
| | Guo et al., 2018 | 16 | 650.69 |
| | Lu et al., 2018 | 28 | 703.93 |
| | Mohammadpour et al., 2019 | 64 | 296.77 |
| | Total | 1238 | |

**Table S2. Post hoc test of Study Groups**

| Sample 1-Sample 2 | Test Statistic | Std. Error | Std. Test Statistic | Adj. Sig.[a] |
|---|---|---|---|---|
| Deng et al., 2021-Chou et al., 2017 | -513.111 | 111.477 | -4.603 | 0.004 |
| Deng et al., 2021-Kim et al., 2021 | -547.283 | 122.117 | -4.482 | 0.007 |
| Deng et al., 2021-Braun et al., 2018 | -548.313 | 107.129 | -5.118 | 0.000 |
| Deng et al., 2021-Zhang et al., 2020 | -553.264 | 119.174 | -4.642 | 0.003 |
| Deng et al., 2021-Kienzle et al., 2017 | -558.676 | 134.799 | -4.145 | 0.034 |
| Deng et al., 2021-Chen et al., 2016 | -566.571 | 123.357 | -4.593 | 0.004 |
| Deng et al., 2021-Lu et al., 2018 | -569.929 | 123.357 | -4.620 | 0.004 |
| Deng et al., 2021-Guo et al., 2016 | -597.281 | 121.022 | -4.935 | 0.001 |
| Deng et al., 2021-Saroj and Rajput, 2018 | -600.583 | 126.403 | -4.751 | 0.002 |
| Deng et al., 2021-Gounani et al., 2018 | -616.615 | 107.422 | -5.740 | 0.000 |
| Deng et al., 2021-Zhao et al., 2019 | 622.250 | 133.241 | 4.670 | 0.003 |
| Deng et al., 2021-Liu et al., 2017 | -627.759 | 122.717 | -5.115 | 0.000 |
| Deng et al., 2021-Wu et al., 2019 | 694.167 | 145.958 | 4.756 | 0.002 |
| Deng et al., 2021-Zhou et al., 2017 | -745.183 | 122.117 | -6.102 | 0.000 |
| Deng et al., 2021-Wang et al., 2019 | -745.708 | 145.958 | -5.109 | 0.000 |
| Deng et al., 2021-Vejdani Noghreiyan et al., 2020 | -784.500 | 190.306 | -4.122 | 0.037 |
| Deng et al., 2021-Ferrauto et al., 2017 | -791.417 | 178.761 | -4.427 | 0.009 |

| | | | | |
|---|---|---|---|---|
| Deng et al., 2021-Paris et al., 2016 | -819.750 | 153.082 | -5.355 | 0.000 |
| Deng et al., 2021-Wright et al., 2021 | 824.167 | 178.761 | 4.610 | 0.004 |
| Deng et al., 2021-Dréau et al., 2016 | -835.679 | 140.649 | -5.942 | 0.000 |
| Deng et al., 2021-Paatero et al., 2017 | -996.750 | 145.958 | -6.829 | 0.000 |
| Ebabe Elle et al., 2016-Fei et al., 2017 | -305.214 | 72.979 | -4.182 | 0.029 |
| Ebabe Elle et al., 2016-Li et al., 2018 | -424.734 | 89.381 | -4.752 | 0.002 |
| Ebabe Elle et al., 2016-Mannerström et al., 2016 | -445.461 | 77.406 | -5.755 | 0.000 |
| Ebabe Elle et al., 2016-Chou et al., 2017 | -505.080 | 75.959 | -6.649 | 0.000 |
| Ebabe Elle et al., 2016-Guo et al., 2018 | -508.656 | 109.468 | -4.647 | 0.003 |
| Ebabe Elle et al., 2016-Yan et al., 2020 | 536.406 | 109.468 | 4.900 | 0.001 |
| Ebabe Elle et al., 2016-Kim et al., 2021 | 539.252 | 90.858 | 5.935 | 0.000 |
| Ebabe Elle et al., 2016-Braun et al., 2018 | -540.282 | 69.420 | -7.783 | 0.000 |
| Ebabe Elle et al., 2016-Zhang et al., 2020 | 545.233 | 86.862 | 6.277 | 0.000 |
| Ebabe Elle et al., 2016-Kienzle et al., 2017 | -550.645 | 107.301 | -5.132 | 0.000 |
| Ebabe Elle et al., 2016-Chen et al., 2016 | -558.540 | 92.518 | -6.037 | 0.000 |
| Ebabe Elle et al., 2016-Lu et al., 2018 | -561.897 | 92.518 | -6.073 | 0.000 |
| Ebabe Elle et al., 2016-Guo et al., 2016 | 589.250 | 89.381 | 6.593 | 0.000 |
| Ebabe Elle et al., 2016-Saroj and Rajput, 2018 | -592.552 | 96.542 | -6.138 | 0.000 |
| Ebabe Elle et al., 2016-Gounani et al., 2018 | -608.583 | 69.872 | -8.710 | 0.000 |
| Ebabe Elle et al., 2016-Zhao et al., 2019 | 614.219 | 105.336 | 5.831 | 0.000 |

| | | | | |
|---|---|---|---|---|
| Ebabe Elle et al., 2016-Liu et al., 2017 | -619.727 | 91.663 | -6.761 | 0.000 |
| Ebabe Elle et al., 2016-Wu et al., 2019 | 686.135 | 121.022 | 5.670 | 0.000 |
| Ebabe Elle et al., 2016-Zhou et al., 2017 | -737.152 | 90.858 | -8.113 | 0.000 |
| Ebabe Elle et al., 2016-Wang et al., 2019 | 737.677 | 121.022 | 6.095 | 0.000 |
| Ebabe Elle et al., 2016-Vejdani Noghreiyan et al., 2020 | 776.469 | 171.927 | 4.516 | 0.006 |
| Ebabe Elle et al., 2016-Ferrauto et al., 2017 | -783.385 | 159.054 | -4.925 | 0.001 |
| Ebabe Elle et al., 2016-Paris et al., 2016 | 811.719 | 129.525 | 6.267 | 0.000 |
| Ebabe Elle et al., 2016-Wright et al., 2021 | 816.135 | 159.054 | 5.131 | 0.000 |
| Ebabe Elle et al., 2016-Dréau et al., 2016 | -827.647 | 114.563 | -7.224 | 0.000 |
| Ebabe Elle et al., 2016-Paatero et al., 2017 | -988.719 | 121.022 | -8.170 | 0.000 |
| Martínez-Carmona et al., 2018-Zhou et al., 2017 | 598.433 | 142.262 | 4.207 | 0.026 |
| Martínez-Carmona et al., 2018-Dréau et al., 2016 | 688.929 | 158.455 | 4.348 | 0.014 |
| Martínez-Carmona et al., 2018-Paatero et al., 2017 | 850.000 | 163.186 | 5.209 | 0.000 |
| Mohammadpour et al., 2019-Mannerström et al., 2016 | 290.719 | 63.202 | 4.600 | 0.004 |
| Mohammadpour et al., 2019-Chou et al., 2017 | 350.338 | 61.421 | 5.704 | 0.000 |
| Mohammadpour et al., 2019-Kim et al., 2021 | 384.510 | 79.107 | 4.861 | 0.001 |
| Mohammadpour et al., 2019-Braun et al., 2018 | 385.539 | 53.121 | 7.258 | 0.000 |
| Mohammadpour et al., 2019-Zhang et al., 2020 | 390.490 | 74.484 | 5.243 | 0.000 |
| Mohammadpour et al., 2019-Kienzle et al., 2017 | 395.903 | 97.551 | 4.058 | 0.049 |
| Mohammadpour et al., 2019-Chen et al., 2016 | 403.798 | 81.008 | 4.985 | 0.001 |
| Mohammadpour et al., 2019-Lu et al., 2018 | 407.155 | 81.008 | 5.026 | 0.000 |
| Mohammadpour et al., 2019-Guo et al., 2016 | 434.508 | 77.406 | 5.613 | 0.000 |
| Mohammadpour et al., 2019-Saroj and Rajput, 2018 | 437.810 | 85.575 | 5.116 | 0.000 |
| Mohammadpour et al., 2019-Gounani et al., 2018 | 453.841 | 53.711 | 8.450 | 0.000 |
| Mohammadpour et al., 2019-Zhao et al., 2019 | 459.477 | 95.386 | 4.817 | 0.001 |

| | | | | |
|---|---|---|---|---|
| Mohammadpour et al., 2019-Liu et al., 2017 | 464.985 | 80.031 | 5.810 | 0.000 |
| Mohammadpour et al., 2019-Wu et al., 2019 | 531.393 | 112.468 | 4.725 | 0.002 |
| Mohammadpour et al., 2019-Zhou et al., 2017 | 582.410 | 79.107 | 7.362 | 0.000 |
| Mohammadpour et al., 2019-Wang et al., 2019 | 582.935 | 112.468 | 5.183 | 0.000 |
| Mohammadpour et al., 2019-Ferrauto et al., 2017 | 628.643 | 152.646 | 4.118 | 0.038 |
| Mohammadpour et al., 2019-Paris et al., 2016 | 656.977 | 121.571 | 5.404 | 0.000 |
| Mohammadpour et al., 2019-Wright et al., 2021 | 661.393 | 152.646 | 4.333 | 0.015 |
| Mohammadpour et al., 2019-Dréau et al., 2016 | 672.905 | 105.486 | 6.379 | 0.000 |
| Mohammadpour et al., 2019-Paatero et al., 2017 | 833.977 | 112.468 | 7.415 | 0.000 |
| You et al., 2016-Paatero et al., 2017 | -796.750 | 178.761 | -4.457 | 0.008 |
| Yang et al., 2019-Gounani et al., 2018 | -347.427 | 69.872 | -4.972 | 0.001 |
| Yang et al., 2019-Zhou et al., 2017 | -475.996 | 90.858 | -5.239 | 0.000 |
| Yang et al., 2019-Paris et al., 2016 | -550.563 | 129.525 | -4.251 | 0.021 |
| Yang et al., 2019-Dréau et al., 2016 | -566.491 | 114.563 | -4.945 | 0.001 |
| Yang et al., 2019-Paatero et al., 2017 | -727.563 | 121.022 | -6.012 | 0.000 |
| Tran et al., 2018-Paatero et al., 2017 | 723.050 | 153.082 | 4.723 | 0.002 |
| Nguyen et al., 2017-Braun et al., 2018 | -265.313 | 61.071 | -4.344 | 0.014 |
| Nguyen et al., 2017-Gounani et al., 2018 | -333.615 | 61.585 | -5.417 | 0.000 |
| Nguyen et al., 2017-Zhou et al., 2017 | 462.183 | 84.651 | 5.460 | 0.000 |
| Nguyen et al., 2017-Paris et al., 2016 | 536.750 | 125.249 | 4.285 | 0.018 |
| Nguyen et al., 2017-Dréau et al., 2016 | -552.679 | 109.705 | -5.038 | 0.000 |
| Nguyen et al., 2017-Paatero et al., 2017 | -713.750 | 116.434 | -6.130 | 0.000 |
| Fei et al., 2017-Braun et al., 2018 | -235.068 | 46.434 | -5.062 | 0.000 |

| | | | | |
|---|---|---|---|---|
| Fei et al., 2017-Gounani et al., 2018 | -303.370 | 47.108 | -6.440 | 0.000 |
| Fei et al., 2017-Liu et al., 2017 | -314.514 | 75.757 | -4.152 | 0.033 |
| Fei et al., 2017-Zhou et al., 2017 | -431.939 | 74.781 | -5.776 | 0.000 |
| Fei et al., 2017-Paris et al., 2016 | 506.505 | 118.801 | 4.263 | 0.020 |
| Fei et al., 2017-Dréau et al., 2016 | -522.434 | 102.282 | -5.108 | 0.000 |
| Fei et al., 2017-Paatero et al., 2017 | -683.505 | 109.468 | -6.244 | 0.000 |
| Maksimović-Ivanić et al., 2019-Paatero et al., 2017 | -676.972 | 157.653 | -4.294 | 0.017 |
| Xie et al., 2016-Paatero et al., 2017 | -646.850 | 153.082 | -4.226 | 0.024 |
| Cheng et al., 2017-Paatero et al., 2017 | -597.125 | 130.549 | -4.574 | 0.005 |
| Li et al., 2018-Paatero et al., 2017 | 563.984 | 121.022 | 4.660 | 0.003 |
| Mannerström et al., 2016-Paatero et al., 2017 | -543.258 | 112.468 | -4.830 | 0.001 |
| Chou et al., 2017-Paatero et al., 2017 | -483.639 | 111.477 | -4.338 | 0.014 |
| Braun et al., 2018-Paatero et al., 2017 | 448.437 | 107.129 | 4.186 | 0.028 |
| Each row tests the null hypothesis that the Sample 1 and Sample 2 distributions are the same. Asymptotic significances (2-sided tests) are displayed. The significance level is 0.05. | | | | |
| [a] Significance values have been adjusted by the Bonferroni correction for multiple tests. | | | | |

**Table S3. Normality Tests for different MSN Sizes**

| | MSN Size | Shapiro-Wilk | | |
|---|---|---|---|---|
| | | Statistic | df | Sig. |
| Viability | under100 | 0.992 | 136 | 0.619 |
| | Between100&200 | 0.801 | 596 | 0.000 |
| | Between200&300 | 0.881 | 309 | 0.000 |
| | Between300&400 | 0.744 | 89 | 0.000 |
| | Between400&500 | 0.933 | 46 | 0.011 |
| | above500 | 0.902 | 64 | 0.000 |

**Table S4. Normality Tests for different viability assay method groups**

| | Viability Assay Method | Shapiro-Wilk | | |
|---|---|---|---|---|
| | | Statistic | df | Sig. |
| Viability | Alamar Blue | 0.962 | 9 | 0.817 |
| | Annexin V/IP | 0.779 | 12 | 0.005 |
| | ATP-assay | 0.823 | 31 | 0.000 |
| | CCK-8 Assay | 0.727 | 246 | 0.000 |
| | CellTiter 96 AQueous Assay | 0.950 | 8 | 0.713 |
| | CellTiterGlo | 0.718 | 41 | 0.000 |
| | FACS | 0.548 | 31 | 0.000 |
| | MTS assay | 0.957 | 30 | 0.257 |
| | MTT | 0.755 | 648 | 0.000 |
| | NRU | 0.865 | 16 | 0.023 |
| | SRB | 0.960 | 6 | 0.816 |
| | WST-1 | 0.940 | 86 | 0.001 |
| | WST-8 | 0.710 | 72 | 0.000 |

**Table S5. Normality Tests for different incubation times**

| | Incubation time | Shapiro-Wilk | | |
|---|---|---|---|---|
| | | Statistic | df | Sig. |
| Viability | 24 h | 0.762 | 567 | 0.000 |
| | 48 h | 0.806 | 467 | 0.000 |
| | 72 h | 0.891 | 183 | 0.000 |
| | | | | |

**Table S6. Normality tests for different MSN zeta-potential**

| | Zeta-potential groups | Shapiro-Wilk | | |
|---|---|---|---|---|
| | | Statistic | df | Sig. |
| Viability | -30 and below | 0.835 | 453 | 0.000 |
| | -30 to -10 | 0.750 | 345 | 0.000 |
| | -10 to +10 | 0.709 | 29 | 0.000 |
| | +10 to +30 | 0.767 | 264 | 0.000 |
| | +30 and above | 0.816 | 149 | 0.000 |

**Table S7. Normality tests for different functionalisation types.**

| Functionalisation Groups | | Shapiro-Wilk | | |
|---|---|---|---|---|
| | | Statistic | df | Sig. |
| Viability | 3- aminopropyl | 0.926 | 22 | 0.103 |
| | F2 | 0.891 | 5 | 0.364 |
| | Ag-Nps | 0.933 | 14 | 0.339 |
| | Amine group (-NH2) | 0.822 | 164 | 0.000 |
| | F5 | 0.912 | 14 | 0.166 |
| | Caffeic acid | 0.817 | 8 | 0.044 |
| | carboxyl functionalised | 0.837 | 138 | 0.000 |
| | F8 | 0.949 | 15 | 0.501 |
| | F9 | 0.960 | 6 | 0.816 |
| | F10 | 0.924 | 24 | 0.072 |

| | | | | |
|---|---|---|---|---|
| | F11 | 0.941 | 24 | 0.175 |
| | F12 | 0.917 | 24 | 0.049 |
| | concanavalin A | 0.950 | 8 | 0.713 |
| | DSPE-PEG2K-FA | 0.891 | 15 | 0.070 |
| | F15 | 0.863 | 10 | 0.084 |
| | Fluorescein PEI-PEG | 0.958 | 17 | 0.599 |
| | F17 | 0.829 | 16 | 0.007 |
| | F18 | 0.911 | 28 | 0.021 |
| | Hyaluronic acid | 0.931 | 16 | 0.249 |
| | F20 | 0.899 | 6 | 0.371 |
| | F21 | 0.913 | 16 | 0.129 |
| | F22 | 0.892 | 14 | 0.085 |
| | none | 0.753 | 463 | 0.000 |
| | OH | 0.867 | 5 | 0.253 |
| | PEG/COOH | 0.948 | 36 | 0.089 |
| | Phenylboronic acid | 0.950 | 12 | 0.632 |
| | F27 | 0.950 | 6 | 0.740 |
| | polyacrylic acid | 0.758 | 24 | 0.000 |
| | polyethyleneimine | 0.813 | 8 | 0.040 |
| | F30 | 0.821 | 8 | 0.048 |
| | Rutin | 0.963 | 8 | 0.842 |
| | F32 | 0.938 | 22 | 0.178 |
| | F33 | 0.973 | 3 | 0.683 |
| | F34 | 1.000 | 3 | 1.000 |
| | F35 | 0.945 | 3 | 0.550 |
| | F36 | 0.882 | 3 | 0.329 |
| | Thiol-modified MSN | 0.695 | 8 | 0.002 |
| | Titanium | 0.967 | 5 | 0.854 |
| | F39 | 0.816 | 14 | 0.008 |
| | F40 | 0.825 | 4 | 0.155 |

**Table S8. Normality test for cell types' viability**

| Cell Type Groups | | Shapiro-Wilk | | |
|---|---|---|---|---|
| | | Statistic | df | Sig. |
| Viability | 3T3 | 0.705 | 6 | 0.007 |
| | 4T1 | 0.954 | 14 | 0.620 |
| | A549 | 0.868 | 6 | 0.217 |
| | A875 | 0.916 | 10 | 0.322 |
| | B16-F10 | 0.967 | 10 | 0.866 |
| | BALB/c 3T3 | 0.876 | 32 | 0.002 |
| | BEAS-2B | 0.831 | 36 | 0.000 |
| | bEND.3 | 0.730 | 6 | 0.013 |
| | C2C12 | 0.896 | 20 | 0.035 |
| | Caco-2 | 0.954 | 40 | 0.103 |
| | CCRF-CEM | 0.981 | 8 | 0.966 |
| | Colon-26 | 0.926 | 4 | 0.571 |
| | COS7 | 0.962 | 10 | 0.805 |
| | Daudi | 0.978 | 10 | 0.950 |
| | DMSCs | 0.902 | 10 | 0.228 |
| | EA.hy926 | 0.898 | 3 | 0.379 |
| | HaCaT | 0.798 | 14 | 0.005 |
| | HaCaT-L | 0.951 | 16 | 0.501 |
| | HEK 293 T | 0.956 | 22 | 0.421 |
| | HEK-293 | 0.756 | 97 | 0.000 |
| | HEK-293TRPA1 | 0.799 | 8 | 0.028 |
| | HEK-293TRPM2 | 0.824 | 8 | 0.051 |
| | HEK-293TRPM8 | 0.880 | 8 | 0.188 |
| | HEK-293TRPV4 | 0.878 | 8 | 0.178 |
| | HeLa | 0.938 | 26 | 0.121 |
| | HEP-G2 | 0.897 | 99 | 0.000 |
| | HFF-1 | 0.837 | 48 | 0.000 |

| | | | | |
|---|---|---|---|---|
| | HFLS | 0.974 | 12 | 0.952 |
| | HL-7702 | 0.804 | 12 | 0.010 |
| | hMSCs | 0.897 | 6 | 0.358 |
| | HOS | 0.963 | 5 | 0.829 |
| | HT-29 | 0.961 | 9 | 0.809 |
| | Huh-7 | 0.982 | 4 | 0.911 |
| | HUVEC | 0.805 | 24 | 0.000 |
| | Jurkat | 0.923 | 10 | 0.384 |
| | L-02 | 0.979 | 54 | 0.440 |
| | L929 | 0.983 | 3 | 0.752 |
| | LNCaP | 0.738 | 12 | 0.002 |
| | LS 174T | 0.980 | 12 | 0.983 |
| | MC3T3-E1 | 0.896 | 4 | 0.410 |
| | MCF-10a | 0.812 | 4 | 0.125 |
| | MCF-7 | 0.838 | 100 | 0.000 |
| | MDA-MB-231 | 0.687 | 32 | 0.000 |
| | MDCK | 0.928 | 6 | 0.565 |
| | MMT | 0.883 | 7 | 0.239 |
| | Mtag | 0.686 | 7 | 0.003 |
| | NCM460 | 0.923 | 3 | 0.464 |
| | NIH-3T3 | 0.783 | 14 | 0.003 |
| | NR8383 | 0.809 | 16 | 0.004 |
| | PC-3 | 0.796 | 12 | 0.008 |
| | Raji | 0.981 | 10 | 0.971 |
| | Ramos (RA1) | 0.909 | 13 | 0.178 |
| | RAW 264.7 | 0.891 | 20 | 0.028 |
| | SCC-7 | 0.891 | 5 | 0.364 |
| | SH-SY5Y | 0.699 | 5 | 0.009 |
| | SK-BR-3 | 0.825 | 4 | 0.155 |
| | SMMC-77721 | 0.939 | 4 | 0.648 |

| | | | | |
|---|---|---|---|---|
| | SW480 | 0.936 | 3 | 0.510 |
| | SW620 | 0.883 | 5 | 0.325 |
| | THP-1 | 0.920 | 36 | 0.013 |
| | TZM-bl | 0.913 | 155 | 0.000 |
| | U937 | 0.930 | 16 | 0.247 |
| | UKRV Mel15a | 0.980 | 6 | 0.953 |
| | WeHi-164 | 0.942 | 11 | 0.542 |

**Table S9. Mean ranks of MSN size groups**

| | MSN Size | N | Mean Rank |
|---|---|---|---|
| Viability | under100 | 136 | 710.14 |
| | Between100&200 | 596 | 663.30 |
| | Between200&300 | 309 | 590.52 |
| | Between300&400 | 89 | 547.81 |
| | Between400&500 | 46 | 592.70 |
| | above500 | 64 | 297.30 |
| | Total | 1240 | |

**Table S10. Post hoc test of MSN size groups**

| Sample 1-Sample 2 | Test Statistic | Std. Error | Std. Test Statistic | Adj. Sig |
|---|---|---|---|---|
| above500-Between300&400 | 250.510 | 58.690 | 4.268 | 0.000 |
| above500-Between200&300 | 293.213 | 49.180 | 5.962 | 0.000 |
| above500-Between400&500 | 295.391 | 69.220 | 4.267 | 0.000 |
| above500-Between100&200 | 365.991 | 47.105 | 7.770 | 0.000 |
| above500-under100 | 412.835 | 54.282 | 7.605 | 0.000 |
| Between300&400-under100 | 162.325 | 48.824 | 3.325 | 0.013 |
| Between200&300-under100 | 119.622 | 36.850 | 3.246 | 0.018 |

**Table S11. Mean ranks of viability assay method Groups**

| | ViabilityAssayMethod | N | Mean Rank |
|---|---|---|---|
| Viability | Alamar Blue | 9 | 519.22 |
| | Annexin V/IP | 12 | 694.17 |
| | ATP-assay | 31 | 689.52 |
| | CCK-8 Assay | 246 | 631.61 |
| | CellTiter 96 AQueous Assay | 8 | 280.88 |
| | CellTiterGlo | 41 | 642.23 |
| | FACS | 31 | 770.16 |
| | MTS assay | 30 | 954.15 |
| | MTT | 648 | 557.41 |
| | NRU | 16 | 684.06 |
| | SRB | 6 | 729.50 |
| | WST-1 | 86 | 759.74 |
| | WST-8 | 72 | 719.08 |
| | Total | 1236 | |

**Table S12. Post hoc test of viability assay method Groups**

| Sample 1-Sample 2 | Test Statistic | Std. Error | Std. Test Statistic | Adj. Sig. |
|---|---|---|---|---|
| CellTiter 96 AQueous Assay-WST-1 | -478.863 | 131.938 | -3.629 | 0.022 |
| CellTiter 96 AQueous Assay-FACS | -489.286 | 141.549 | -3.457 | 0.043 |
| CellTiter 96 AQueous Assay-MTS assay | -673.275 | 142.033 | -4.740 | 0.000 |
| MTT-WST-8 | -161.674 | 44.342 | -3.646 | 0.021 |
| MTT-WST-1 | -202.329 | 40.965 | -4.939 | 0.000 |
| MTT-MTS assay | 396.740 | 66.660 | 5.952 | 0.000 |

| | | | | |
|---|---|---|---|---|
| CCK-8 Assay-MTS assay | -322.544 | 69.028 | -4.673 | 0.000 |
| CellTiterGlo-MTS assay | -311.918 | 85.759 | -3.637 | 0.022 |

**Table S13. Mean ranks of incubation time groups**

| | Incubation time | N | Mean Rank |
|---|---|---|---|
| Viability | 24 h | 567 | 631.26 |
| | 48 h | 467 | 565.56 |
| | 72 h | 183 | 650.88 |
| | Total | 1217 | |

**Table S14. Post hoc test of incubation time groups**

| Sample 1-Sample 2 | Test Statistic | Std. Error | Std. Test Statistic | Adj. Sig |
|---|---|---|---|---|
| 48 h – 24 h | 65.701 | 21.963 | 2.991 | 0.008 |
| 48 h – 72 h | -85.316 | 30.651 | -2.783 | 0.016 |
| 24 h – 72 h | -19.615 | 29.881 | -.656 | 1.000 |

**Table S15. Mean ranks of MSN zeta-potential Groups**

| | Zeta-potential groups | N | Mean Rank |
|---|---|---|---|
| Viability | -30 and below | 453 | 637.79 |
| | -30 to -10 | 345 | 603.58 |
| | -10 to +10 | 29 | 939.62 |
| | +10 to +30 | 264 | 611.35 |
| | +30 and above | 149 | 561.20 |
| | Total | 1240 | |

**Table S16. Post hoc test of MSNs' zeta-potential Groups**

| Sample 1 - Sample 2 | Test Statistic | Std. Error | Std. Test Statistic | Adj. Sig |
|---|---|---|---|---|
| +30 and above - (-10 to +10) | 378.419 | 72.681 | 5.207 | 0.000 |
| -30 to -10 - (-10 to +10) | -336.041 | 69.236 | -4.854 | 0.000 |
| +10 to +30 - (-10 to +10) | 328.270 | 70.055 | 4.686 | 0.000 |
| -30 and below – (-10 to +10) | -301.827 | 68.593 | -4.400 | 0.000 |

**Table S17. Shapiro Wilk's Test of normality for functionalised and non-functionalised MSN groups in the dataset**

| | Functionalised or Non-Functionalised | Shapiro-Wilk | | |
|---|---|---|---|---|
| | | Statistic | df | Sig. |
| Viability | No Surface Functionalization | 0.753 | 463 | 0.000 |
| | Surface Functionalised | 0.820 | 777 | 0.000 |

**Table S18. Mean ranks of Functionalization Groups**

| | Functionalization Groups | N | Mean Rank |
|---|---|---|---|
| Viability | 3- aminopropyl | 22 | 416.66 |
| | F2 | 5 | 604.80 |
| | Ag-Nps | 14 | 509.79 |
| | Amine group (-NH2) | 164 | 600.83 |
| | F5 | 14 | 899.86 |
| | Caffeic acid | 8 | 78.50 |

| | | | |
|---|---|---|---|
| | carboxyl functionalised | 138 | 791.02 |
| | F8 | 15 | 811.73 |
| | F9 | 6 | 731.00 |
| | F10 | 24 | 412.33 |
| | F11 | 24 | 514.33 |
| | F12 | 24 | 528.56 |
| | concanavalin A | 8 | 281.25 |
| | DSPE-PEG2K-FA | 15 | 751.93 |
| | F15 | 10 | 408.50 |
| | Fluorescein PEI-PEG | 17 | 693.68 |
| | F17 | 16 | 668.94 |
| | F18 | 28 | 701.57 |
| | Hyaluronic acid | 16 | 695.34 |
| | F20 | 6 | 643.33 |
| | F21 | 16 | 651.44 |
| | F22 | 14 | 681.43 |
| | none | 463 | 570.02 |
| | OH | 5 | 910.60 |
| | PEG/COOH | 36 | 688.26 |
| | Phenylboronic acid | 12 | 829.17 |
| | F27 | 6 | 499.58 |
| | polyacrylic acid | 24 | 735.58 |
| | polyethyleneimine | 8 | 281.75 |
| | F30 | 8 | 475.63 |
| | Rutin | 8 | 268.06 |
| | F32 | 22 | 418.39 |
| | F33 | 3 | 1231.17 |
| | F34 | 3 | 830.67 |
| | F35 | 3 | 1229.17 |
| | F36 | 3 | 1235.67 |

| | | | |
|---|---|---|---|
| | Thiol-modified MSN | 8 | 739.00 |
| | Titanium | 5 | 919.50 |
| | F39 | 14 | 970.68 |
| | F40 | 4 | 700.88 |
| | Total | 1239 | |

**Table S19. Post hoc test of functionalization Groups.** [a] Significance values have been adjusted by the Bonferroni correction for multiple tests

| Sample 1-Sample 2 | Test Statistic | Std. Error | Std. Test Statistic | Adj. Sig.[a] |
|---|---|---|---|---|
| Caffeic acid-Amine group (-NH2) | 522.332 | 129.554 | 4.032 | 0.043 |
| Caffeic acid-PEG/COOH | -609.764 | 139.857 | -4.360 | 0.010 |
| Caffeic acid-Fluorescein PEI-PEG | -615.176 | 153.410 | -4.010 | 0.047 |
| Caffeic acid-F18 | -623.071 | 143.444 | -4.344 | 0.011 |
| Caffeic acid-polyacrylic acid | -657.083 | 146.076 | -4.498 | 0.005 |
| Caffeic acid-DSPE-PEG2K-FA | -673.433 | 156.649 | -4.299 | 0.013 |
| Caffeic acid-carboxyl functionalised | -712.518 | 130.120 | -5.476 | 0.000 |
| Caffeic acid-F8 | -733.233 | 156.649 | -4.681 | 0.002 |
| Caffeic acid-Phenylboronic acid | -750.667 | 163.318 | -4.596 | 0.003 |
| Caffeic acid-F5 | 821.357 | 158.583 | 5.179 | 0.000 |
| Caffeic acid-OH | -832.100 | 203.984 | -4.079 | 0.035 |
| Caffeic acid-Titanium | -841.000 | 203.984 | -4.123 | 0.029 |
| Caffeic acid-F39 | -892.179 | 158.583 | -5.626 | 0.000 |
| Caffeic acid-F35 | -1150.667 | 242.239 | -4.750 | 0.002 |
| Caffeic acid-F33 | -1152.667 | 242.239 | -4.758 | 0.002 |
| Caffeic acid-F36 | -1157.167 | 242.239 | -4.777 | 0.001 |
| Rutin-carboxyl functionalised | 522.956 | 130.120 | 4.019 | 0.046 |
| Rutin-F39 | -702.616 | 158.583 | -4.431 | 0.007 |
| concanavalin A-F39 | -689.429 | 158.583 | -4.347 | 0.011 |
| polyethyleneimine-F39 | -688.929 | 158.583 | -4.344 | 0.011 |
| F10-carboxyl functionalised | 378.685 | 79.135 | 4.785 | 0.001 |

| | | | | |
|---|---|---|---|---|
| F10-F5 | 487.524 | 120.331 | 4.052 | 0.040 |
| F10-F39 | -558.345 | 120.331 | -4.640 | 0.003 |
| 3- aminopropyl-carboxyl functionalised | -374.359 | 82.142 | -4.557 | 0.004 |
| 3- aminopropyl-F39 | -554.019 | 122.329 | -4.529 | 0.005 |
| F32-carboxyl functionalised | 372.632 | 82.142 | 4.536 | 0.004 |
| F32-F39 | -552.292 | 122.329 | -4.515 | 0.005 |
| none-carboxyl functionalised | 221.002 | 34.703 | 6.368 | 0.000 |
| none-F39 | -400.662 | 97.064 | -4.128 | 0.029 |
| Amine group (-NH2)-carboxyl functionalised | -190.186 | 41.333 | -4.601 | 0.003 |

**Table S20. Mean ranks of cell types Groups**

| | Cell Type | N | Mean Rank |
|---|---|---|---|
| Viability | 3T3 | 6 | 773.75 |
| | 4T1 | 14 | 655.07 |
| | A549 | 6 | 760.08 |
| | A875 | 10 | 622.50 |
| | B16-F10 | 10 | 758.05 |
| | BALB/c 3T3 | 32 | 459.77 |
| | BEAS-2B | 36 | 758.47 |
| | bEND.3 | 6 | 945.92 |
| | C2C12 | 20 | 970.53 |
| | Caco-2 | 40 | 291.44 |
| | CCRF-CEM | 8 | 395.00 |
| | Colon-26 | 4 | 703.00 |
| | COS7 | 10 | 529.70 |
| | Daudi | 10 | 956.50 |

| | | | |
|---|---|---|---|
| | DMSCs | 10 | 955.45 |
| | EA.hy926 | 3 | 210.33 |
| | HaCaT | 14 | 717.00 |
| | HaCaT-L | 16 | 161.44 |
| | HEK 293 T | 22 | 511.89 |
| | HEK-293 | 97 | 569.76 |
| | HEK-293TRPA1 | 8 | 419.81 |
| | HEK-293TRPM2 | 8 | 290.50 |
| | HEK-293TRPM8 | 8 | 304.00 |
| | HEK-293TRPV4 | 8 | 303.13 |
| | HeLa | 26 | 640.67 |
| | HEP-G2 | 99 | 665.27 |
| | HFF-1 | 48 | 732.72 |
| | HFLS | 12 | 678.00 |
| | HL-7702 | 12 | 674.46 |
| | hMSCs | 6 | 927.08 |
| | HOS | 5 | 305.40 |
| | HT-29 | 9 | 747.61 |
| | Huh-7 | 4 | 1052.88 |
| | HUVEC | 24 | 793.69 |
| | Jurkat | 10 | 779.20 |
| | L-02 | 54 | 544.61 |
| | L929 | 3 | 359.00 |
| | LNCaP | 12 | 733.54 |
| | LS 174T | 12 | 211.42 |
| | MC3T3-E1 | 4 | 384.25 |
| | MCF-10a | 4 | 633.50 |
| | MCF-7 | 100 | 576.49 |
| | MDA-MB-231 | 32 | 567.77 |
| | MDCK | 6 | 959.83 |

| | | | |
|---|---|---|---|
| | MMT | 7 | 883.64 |
| | Mtag | 7 | 1059.43 |
| | NCM460 | 3 | 799.00 |
| | NIH-3T3 | 14 | 693.50 |
| | NR8383 | 16 | 472.06 |
| | PC-3 | 12 | 738.54 |
| | Raji | 10 | 906.65 |
| | Ramos (RA1) | 13 | 414.69 |
| | RAW 264.7 | 20 | 387.98 |
| | SCC-7 | 5 | 605.00 |
| | SH-SY5Y | 5 | 275.60 |
| | SK-BR-3 | 4 | 701.38 |
| | SMMC-77721 | 4 | 829.50 |
| | SW480 | 3 | 488.33 |
| | SW620 | 5 | 530.60 |
| | THP-1 | 36 | 538.22 |
| | TZM-bl | 155 | 683.53 |
| | U937 | 16 | 962.75 |
| | UKRV Mel15a | 6 | 738.25 |
| | WeHi-164 | 11 | 669.82 |
| | Total | 1240 | |

**Table S21. Post hoc test of cell type Groups**

| Sample 1-Sample 2 | Test Statistic | Std. Error | Std. Test Statistic | Adj. Sig |
|---|---|---|---|---|
| HaCaT-L-HEK-293 | -408.325 | 96.627 | -4.226 | 0.048 |
| HaCaT-L-MCF-7 | -415.048 | 96.421 | -4.305 | 0.034 |
| HaCaT-L-HEP-G2 | -503.830 | 96.488 | -5.222 | 0.000 |

| | | | | |
|---|---|---|---|---|
| HaCaT-L-TZM-bl | -522.095 | 94.032 | -5.552 | 0.000 |
| HaCaT-L-HaCaT | 555.563 | 131.051 | 4.239 | 0.045 |
| HaCaT-L-HFF-1 | -571.281 | 103.375 | -5.526 | 0.000 |
| HaCaT-L-PC-3 | -577.104 | 136.752 | -4.220 | 0.049 |
| HaCaT-L-BEAS-2B | 597.035 | 107.596 | 5.549 | 0.000 |
| HaCaT-L-Jurkat | -617.763 | 144.355 | -4.279 | 0.038 |
| HaCaT-L-HUVEC | -632.250 | 115.576 | -5.470 | 0.000 |
| HaCaT-L-MMT | -722.205 | 162.278 | -4.450 | 0.017 |
| HaCaT-L-Raji | -745.213 | 144.355 | -5.162 | 0.000 |
| HaCaT-L-hMSCs | -765.646 | 171.427 | -4.466 | 0.016 |
| HaCaT-L-bEND.3 | 784.479 | 171.427 | 4.576 | 0.010 |
| HaCaT-L-DMSCs | 794.013 | 144.355 | 5.500 | 0.000 |
| HaCaT-L-Daudi | 795.063 | 144.355 | 5.508 | 0.000 |
| HaCaT-L-MDCK | -798.396 | 171.427 | -4.657 | 0.006 |
| HaCaT-L-U937 | -801.313 | 126.607 | -6.329 | 0.000 |
| HaCaT-L-C2C12 | 809.088 | 120.110 | 6.736 | 0.000 |
| HaCaT-L-Huh-7 | -891.438 | 200.184 | -4.453 | 0.017 |
| HaCaT-L-Mtag | -897.991 | 162.278 | -5.534 | 0.000 |
| LS 174T-TZM-bl | -472.116 | 107.302 | -4.400 | 0.022 |
| LS 174T-HFF-1 | 521.302 | 115.576 | 4.510 | 0.013 |
| LS 174T-BEAS-2B | 547.056 | 119.367 | 4.583 | 0.009 |
| LS 174T-HUVEC | 582.271 | 126.607 | 4.599 | 0.009 |

| | | | | |
|---|---|---|---|---|
| LS 174T-Raji | -695.233 | 153.329 | -4.534 | 0.012 |
| LS 174T-DMSCs | 744.033 | 153.329 | 4.853 | 0.002 |
| LS 174T-Daudi | 745.083 | 153.329 | 4.859 | 0.002 |
| LS 174T-U937 | -751.333 | 136.752 | -5.494 | 0.000 |
| LS 174T-C2C12 | 759.108 | 130.760 | 5.805 | 0.000 |
| LS 174T-Mtag | -848.012 | 170.310 | -4.979 | 0.001 |
| HEK-293TRPM2-U937 | -672.250 | 155.062 | -4.335 | 0.029 |
| HEK-293TRPM2-C2C12 | 680.025 | 149.804 | 4.539 | 0.011 |
| Caco-2-MCF-7 | -285.048 | 66.994 | -4.255 | 0.042 |
| Caco-2-HEP-G2 | -373.830 | 67.091 | -5.572 | 0.000 |
| Caco-2-TZM-bl | -392.095 | 63.508 | -6.174 | 0.000 |
| Caco-2-HFF-1 | -441.281 | 76.665 | -5.756 | 0.000 |
| Caco-2-BEAS-2B | 467.035 | 82.268 | 5.677 | 0.000 |
| Caco-2-HUVEC | -502.250 | 92.461 | -5.432 | 0.000 |
| Caco-2-Raji | -615.213 | 126.607 | -4.859 | 0.002 |
| Caco-2-DMSCs | -664.013 | 126.607 | -5.245 | 0.000 |
| Caco-2-Daudi | -665.063 | 126.607 | -5.253 | 0.000 |
| Caco-2-MDCK | -668.396 | 156.775 | -4.263 | 0.041 |
| Caco-2-U937 | -671.313 | 105.927 | -6.337 | 0.000 |
| Caco-2-C2C12 | 679.088 | 98.070 | 6.925 | 0.000 |

| | | | | |
|---|---|---|---|---|
| Caco-2-Mtag | -767.991 | 146.715 | -5.235 | 0.000 |
| HEK-293TRPV4-U937 | -659.625 | 155.062 | -4.254 | 0.042 |
| HEK-293TRPV4-C2C12 | 667.400 | 149.804 | 4.455 | 0.017 |
| HEK-293TRPM8-U937 | -658.750 | 155.062 | -4.248 | 0.043 |
| HEK-293TRPM8-C2C12 | 666.525 | 149.804 | 4.449 | 0.017 |
| RAW 264.7-U937 | -574.775 | 120.110 | -4.785 | 0.003 |
| RAW 264.7-C2C12 | 582.550 | 113.241 | 5.144 | 0.001 |
| RAW 264.7-Mtag | 671.454 | 157.261 | 4.270 | 0.039 |
| Ramos (RA1)-C2C12 | 555.833 | 127.578 | 4.357 | 0.027 |
| BALB/c 3T3-U937 | -502.984 | 109.645 | -4.587 | 0.009 |
| BALB/c 3T3-C2C12 | -510.759 | 102.074 | -5.004 | 0.001 |
| THP-1-C2C12 | 432.303 | 99.869 | 4.329 | 0.030 |
| L-02-C2C12 | 425.914 | 93.736 | 4.544 | 0.011 |
| HEK-293-C2C12 | 400.762 | 87.942 | 4.557 | 0.010 |
| MCF-7-C2C12 | 394.040 | 87.716 | 4.492 | 0.014 |
